# A multi-breed reference panel and additional rare variants maximize imputation accuracy in cattle

**DOI:** 10.1186/s12711-019-0519-x

**Published:** 2019-12-26

**Authors:** Troy N. Rowan, Jesse L. Hoff, Tamar E. Crum, Jeremy F. Taylor, Robert D. Schnabel, Jared E. Decker

**Affiliations:** 1 Division of Animal Sciences, University of Missouri, Columbia, MO 65211 USA; 2 Informatics Institute, University of Missouri, Columbia, MO 65211 USA

## Abstract

**Background:**

During the last decade, the use of common-variant array-based single nucleotide polymorphism (SNP) genotyping in the beef and dairy industries has produced an astounding amount of medium-to-low density genomic data. Although low-density assays work well in the context of genomic prediction, they are less useful for detecting and mapping causal variants and the effects of rare variants are not captured. The objective of this project was to maximize the accuracies of genotype imputation from medium- and low-density assays to the marker set obtained by combining two high-density research assays (~ 850,000 SNPs), the Illumina BovineHD and the GGP-F250 assays, which contains a large proportion of rare and potentially functional variants and for which the assay design is described here. This 850 K SNP set is useful for both imputation to sequence-level genotypes and direct downstream analysis.

**Results:**

We found that a large multi-breed composite imputation reference panel that includes 36,131 samples with either BovineHD and/or GGP-F250 genotypes significantly increased imputation accuracy compared with a within-breed reference panel, particularly at variants with low minor allele frequencies. Individual animal imputation accuracies were maximized when more genetically similar animals were represented in the composite reference panel, particularly with complete 850 K genotypes. The addition of rare variants from the GGP-F250 assay to our composite reference panel significantly increased the imputation accuracy of rare variants that are exclusively present on the BovineHD assay. In addition, we show that an assay marker density of 50 K SNPs balances cost and accuracy for imputation to 850 K.

**Conclusions:**

Using high-density genotypes on all available individuals in a multi-breed reference panel maximized imputation accuracy for tested cattle populations. Admixed animals or those from breeds with a limited representation in the composite reference panel were still imputed at high accuracy, which is expected to further increase as the reference panel expands. We anticipate that the addition of rare variants from the GGP-F250 assay will increase the accuracy of imputation to sequence level.

## Background

High-density single nucleotide polymorphism (SNP) genotyping has driven rapid improvements in rates of genetic progress in livestock populations [1–3]. To increase the predictive capabilities of genomic prediction models further, the discovery of functional variants has become increasingly important. Although many large-effect or Mendelian variants that control important phenotypes in cattle have been identified [4–8], the identification of moderate and small effect quantitative trait nucleotides (QTN) and other causal variants has proven challenging [9–11]. Early genome-wide association studies (GWAS) that focused on the detection of these variants were often forced to choose between the density of the SNP array (number of SNPs genotyped) and statistical power (number of individuals genotyped). Imputation, the use of statistical models, and a reference set of haplotypes to infer missing genotypes, allows researchers to genotype large numbers of individuals at relatively low-density and impute their genotypes to high-density or even millions of SNPs from whole-genome resequencing data [12–14].

Low- to medium-density common variant SNP assays are widely used for genetic evaluation in both beef and dairy cattle. Since the development of the BovineSNP50 (SNP50) BeadChip (Illumina, San Diego, CA) [15] in 2008 and the BovineHD (Illumina, San Diego, CA) array in 2009, more than 3 million dairy cattle in the United States alone have been genotyped using SNP assays that are derived from these progenitor assays [16]. Decker [14] noted the value of these commercially-generated datasets for uses beyond genetic prediction. Although lower-density assays work well for genomic prediction [16–18], the effects due to rare variants are not captured and they have a low resolution for the detection of quantitative trait loci (QTL) or causal variants. High-quality imputation allows these datasets to be used to their full potential [19–21]. Seabury et al. [15] found that similar trait heritabilities were obtained with 50 K common variant genotypes and 778 K common variant imputed genotypes, but that the former were less powerful for QTL detection. Imputed 778 K genotypes identified 14 putative large effect QTL that were not identified using 50 K genotypes. Using these large publicly-funded or commercially-generated datasets imputed to high-resolution marker densities will increase prediction accuracies, aid in the detection of causal variants, and ultimately increase selection response in cattle [21–24].

To use these large datasets to their full potential, the accuracy of imputation must be maximized. The most accurate imputation software packages for cattle [19, 25] were typically developed for human studies that were aimed at imputing from a high-density genotype panel to full-genome sequence. As a result, using these programs to impute genotypes directly from low-density to full-genome sequence, even in cattle breeds with high levels of linkage disequilibrium (LD), has been less accurate [21]. A “two-step” imputation strategy, first from a low-density assay (8 K to 70 K variants) to a high-density assay (> 700 K variants) and then from imputed high-density to the sequence level was more accurate than genotypes imputed in “one-step” from low-density to full-genome sequence in both cattle and humans [26, 27]. In this study, we consider the first part of the “two-step” imputation processes, because the produced genotypes can be used as input for imputation to full-genome sequence or as an endpoint for a variety of downstream analyses. Regardless of its use, maximizing the accuracy of imputation to high-density genotypes is essential to the success of both approaches.

Initially, SNP assays for cattle were designed with common, evenly spaced markers that would presumably be in LD with causal variants [15]. Whereas these assays have performed well in genomic prediction applications, there is growing interest in including rare variants into predictions [12, 21, 24, 28]. Imputation accuracy has been shown to decline rapidly as minor allele frequencies (MAF) of SNPs decrease, thus increasing the confidence in the imputation of genotypes for rare variants has become a priority. In addition, most studies on optimizing imputation have focused on the imputation of genotypes for purebred animals using closely-related individuals from the same breed. As large numbers of genotypes for unpedigreed crossbred animals have become available, it is necessary to re-evaluate strategies for genotype imputation in these datasets.

This study focuses on maximizing imputation accuracy from several commercially available low-density common variant SNP genotyping assays to a set of high-density variants (850 K), many of which are rare and potentially functional. We test the effectiveness of a large, multi-breed composite reference panel for imputation in several beef and dairy cattle populations that are genotyped with several commercially available common variant SNP genotyping assays. We use both well-established and novel measures of imputation accuracy to categorize precisely the causes of imputation errors. These metrics provide insights for interpretation of imputation performance and define situations in which researchers should be cautious when using imputed variants. In addition, we explore how the starting chip density impacts the accuracy of imputation to 850 K variants. Finally, we introduce and describe the design of the GGP-F250 functional genotyping assay. The GGP-F250 is a tool not only for genotyping numerous functional variants but also for increasing the imputation accuracy of rare variants.

## Methods

**Fig. 1:**
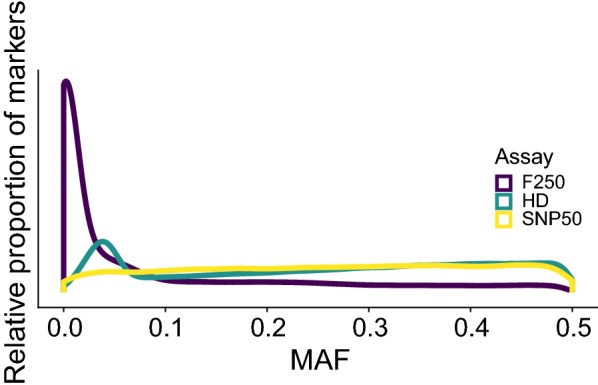
Minor allele frequency spectra for three commercially available assays with different marker densities. Density plot of minor allele frequencies for the SNP50 (yellow), F250 (purple), and HD (green) assays

To identify the best practices for achieving imputation accuracies that approach the error rates of modern SNP genotyping arrays, we compared the impact of altering reference panels and marker numbers in the starting assay when imputing genotypes to the level of the combined Illumina BovineHD (Illumina, San Diego, CA) and GeneSeek Genomic Profiler F250 (GeneSeek. Lincoln, NE) referred to herein as the HD and F250 assays, respectively. The HD assay contains 777,962 evenly spaced variants that have relatively high MAF across many breeds of cattle common to North America. The F250 assay contains 227,234 markers, of which 31,392 are present on the HD assay and included in the assay design for use in imputation, and another 195,842 potentially functional markers, many of which are rare (MAF < 0.1). Due to these rare alleles, the MAF distribution for the F250 assay is more similar to the site frequency spectrum of the bovine genome (Fig. 1). Details on the design of the F250 assay are in Additional file 1: Tables S1–S4. In this study, we used 2718 animals that were genotyped with both the F250 and HD assays, and 25,772 animals that were genotyped with only the F250, and 7218 animals genotyped with only the HD assay.

### Quality control and filtering

Prior to sub-setting and masking genotypes for testing, we used the PLINK1.9 software [29] to filter variants and individuals. The SNP positions were based on the ARS-UCD1.2 bovine reference genome assembly [30]. Non-autosomal variants were removed from the data. Variants and individuals with call rates lower than 0.90 were removed from the testing and reference datasets. Because many of the F250 variants are rare, no MAF filter was applied to any of the SNP arrays. Due to the diverse breed composition of the dataset, no Hardy–Weinberg equilibrium filter was applied. PLINK was used to estimate MAF in the filtered dataset for use in all downstream analyses. Two animals were removed due to low genotype call rates. The numbers of remaining variants after filtering for each of the assays in the masked testing set are in Table 1.

**Table 1:** Variant counts for masked genotypes of 307 testing individuals used in this analysis before and after filtering

Assay	Starting assay density	Filtered density
GGP-ULD	8672	6394
GGP-LD	26,504	16,854
SNP50	58,336	44,366
GGP-90KT	76,999	70,581
GGP-HD	139,977	125,446
GGP-F250	227,234	201,236
HD	777,962	753,715

### Creating the imputation test set

To test the accuracy of imputation, PLINK1.9 [29] was used to down-sample genotypes for 307 animals with both HD and F250 genotypes to the densities found on several commonly used commercial genotyping arrays: SNP50 and GGP-LD, GGP-90KT, GGP-HDv3, and GGP-ULD (all from GeneSeek, Lincoln, NE), which were then imputed to the combined high-density research chips (~ 850 K SNPs). The process of sampling and masking testing genotypes is described visually in Additional file 2: Figure S1. All tested commercial assays possess SNPs that are largely derived from the SNP set of the HD assay (see Additional file 3: Table S5).

A maximum of 50 individuals per breed that were genotyped with both the HD and F250 assays, were randomly chosen and masked to represent various commercial chip densities for testing imputation accuracy (Table 2). All test set individuals had their breed-composition estimated by the CRUMBLER pipeline [31]. To avoid depleting the reference panel of breeds with small numbers of research assay genotypes, no more than 50% of a breed’s F250 or HD genotyped animals were removed for testing. The remainder of the HD and F250 genotypes were used in the composite reference panel (Table 2). Due to the unequal representation of breeds in the test dataset, we created three separate datasets for testing different aspects of our imputation pipeline. The first dataset, ALL, used all 307 masked individuals that passed genotype call rate filtering. Because some of the indicine breeds used in our testing dataset were not adequately represented in the imputation reference panel, or their testing dataset sample sizes were not sufficiently large to draw meaningful conclusions, we created a test dataset, TAUR, which comprised only *Bos taurus* animals, i.e. 281 Angus, Gelbvieh, Hereford, Holstein, Limousin and Simmental individuals. Finally, we used a test dataset, GEL that included 49 Gelbvieh individuals, to compare the accuracy of a within-breed imputation reference to the composite reference.

**Table 2:** Breed representation of the test set and composite reference (CR) panel after filtering

Breed	Number of testing individuals	HD and F250^a, b^	HD^b, c^	F250^b, c^	HD and F250^a^ (%)	HD (%)	F250 (%)
Holstein	50	1932	3170	1944	80.13	32.92	6.90
Gelbvieh	49	257	265	514	10.66	2.75	1.82
Angus	50	132	2067	14,454	5.47	21.47	51.29
Simmental	50	67	427	1759	2.78	4.43	6.24
Brahman	5	7	25	632	0.29	0.26	2.24
Romagnola	4	4	11	4	0.17	0.11	0.01
Nelore	4	3	855	4	0.12	8.88	0.01
Jersey	4	3	21	5	0.12	0.22	0.02
Gir	5	3	10	6	0.12	0.10	0.02
N’Dama	4	3	7	4	0.12	0.07	0.01
Brangus	0	0	990	1603	0.00	10.28	5.69
Hereford	44	0	569	1834	0.00	5.91	6.51
Mixed/crossbred	0	0	419	2830	0.00	4.35	10.04
Red Angus	0	0	253	1905	0.00	2.63	6.76
Limousin	38	0	215	142	0.00	2.23	0.50
Shorthorn	0	0	136	218	0.00	1.41	0.77
Charolais	0	0	125	284	0.00	1.30	1.01
Santa Gertrudis	0	0	23	11	0.00	0.24	0.04
Japanese Black	0	0	19	0	0.00	0.20	0.00
Brown Swiss	0	0	15	0	0.00	0.16	0.00
Norwegian Red	0	0	5	0	0.00	0.05	0.00
Chianina	0	0	2	1	0.00	0.02	0.00
Piedmontese	0	0	0	9	0.00	0.00	0.03
Braunvieh	0	0	0	7	0.00	0.00	0.02
Guernsey	0	0	0	7	0.00	0.00	0.02
Beefmaster	0	0	0	3	0.00	0.00	0.01
Sheko	0	0	0	2	0.00	0.00	0.01
Maine Anjou	0	0	0	1	0.00	0.00	0.00
Total	307	2411	9629	28,183	100	100	100

^a^Animals genotyped with both the HD and F250

^b^Number of individuals in CR remaining after 307 testing individuals were removed

^c^Includes individuals genotyped on both HD and F250

### Phasing and imputation

#### Building phasing and imputation reference panels

After removing 307 individuals for testing, the remaining 28,183 F250 and 9629 HD genotyped reference individuals (Table 2) were merged in PLINK and then phased with Eagle 2.4 [32]. Missing genotypes inferred by Eagle were removed with the bcftools program [33] such that only the phased, directly genotyped markers remained.

The within-breed imputation reference panel consisted of 265 and 514 Gelbvieh individuals that were genotyped with the HD and F250 assays, respectively. These reference individuals had their genotypes merged and phased, and the inferred genotypes were removed separately for each assay. Reciprocal F250/HD imputation analyses performed with Minimac3 were used to fill in missing genotypes in the reference panel.

#### Phasing and imputation

Reference-based phasing was performed for 307 individuals with masked genotypes in Eagle using 9629 individuals with pre-phased HD assay genotypes as the reference haplotypes. To perform “one-round” imputation, phased assays were imputed against the complete imputed 850 K SNP composite reference panel using Minimac3 [34]. The reference panel for the “one-round” imputation process was created by imputing missing HD markers for individuals genotyped on the F250 assay, and missing genotypes for F250 markers for individuals genotyped on the HD assay with Minimac3 (see Additional file 4: Figure S2). Here, the reference panel contained both observed and imputed genotypes.

For “two-round” imputation, two separate imputation steps were performed to reach the 850 K SNP density (see Additional file 4: Figure S2). In each step, only observed genotypes served as HD and F250 references, respectively (no imputed genotypes in reference). First, the testing individuals with masked and phased genotypes were imputed to HD density (759,329 SNPs), and then a second imputation step was performed that inferred genotypes for markers present on the F250, but not on the HD (122,181 SNPs) assay. Both imputation methods resulted in a total number of 835,947 variants, of which 835,926 segregated in the “one-round” CR panel and 835,933 in the “two-round” CR panel.

For the within-breed imputation, 49 Gelbvieh animals, all of which were present in the multi-breed testing set, which had been genotyped with both the F250 and HD assays, were masked to SNP50 density. Genotypes for these individuals were phased using Eagle along with 1113 additional Gelbvieh individuals genotyped with the SNP50 assay. This is representative of phasing strategies that involve a large number of individuals that have been genotyped using lower density assays. Phased genotypes were imputed against the breed-specific Gelbvieh reference (BR) panel.

### Measures of imputation accuracy

Imputation accuracy was measured for both individuals and variants within each imputation scenario. By coding alternate allele counts as 0, 1, and 2 (for *AA*, *AB*, and *BB* genotypes, respectively), both Pearson’s correlation coefficient (*r*) and count-based metrics could be used to evaluate the imputation accuracy for each variant and individual. Pearson’s correlation coefficients for individuals were calculated in two ways. First using unscaled, raw genotype values and then using genotype values centered by the variant’s MAF in the entire set of research assays (all HD, F250, reference and testing). To center genotypes, twice the variant’s MAF was subtracted from the raw genotype value. For both methods, *r* values were calculated and compared.

Although simple concordance (i.e., “correct/incorrect”) measures of accuracy are valuable, they overestimate the quality of imputation at low MAF and are ambiguous as to the nature of the error that created an incorrectly imputed genotype. Rather than concordance rate, an imputation quality score (IQS) [35] was calculated for each variant. The IQS calculates concordances that are adjusted for the chance that an imputed genotype could be correctly guessed. This statistic provides similar conclusions to correlation coefficients for most markers, but it estimates more robustly imputation quality for variants with low MAF [35]. Since Pearson’s correlation coefficients cannot be calculated in the absence of variation, a marker that appears fixed with the reference in the true set of genotypes, but contains an alternate allele when imputed, cannot have an *r* computed, but can have an IQS. This idea also applies in all cases when a marker is fixed in the true or imputed set, but not in the other. IQS allows us to identify all of these specific error types, and thus provides a more complete account of imputation accuracy.

In addition to the IQS, the exact nature of each error was catalogued and tallied for each individual and variant. This allowed the errors to be categorized as either false heterozygotes (genotyped *AA* or *BB* imputed as *AB*), false homozygotes (genotyped *AB* imputed as *AA* or *BB*) or completely discordant (*BB* imputed as *AA* or vice versa). These more detailed error descriptions, in conjunction with MAF, genome position, and assay-of-origin information, allow for a detailed analysis of how these factors influence imputation accuracy to 850 K in each scenario.

To approximate how well represented each individual was in the composite reference, we created a standardized genomic relationship matrix (GRM) as described in [36] using the GEMMA software [37]. The resulting values provide quantitative measures of how far each individual is diverged from the members of the composite reference panel, i.e. larger values indicate that the individuals are more closely related to the animals in the reference panel. To observe the impact of within-breed genetic similarity on imputation accuracy, we created four breed-specific standardized GRM using test individuals and individuals in the reference with more than 50% Angus (number in test = 50, number in reference = 15,013), Holstein (number in test = 50, number of reference = 5127), Gelbvieh (number of testing = 49, number of reference = 470), and Brahman/Nelore (number of testing = 50, number of reference = 2043) ancestry reported from the CRUMBLER pipeline [31]. Row means were calculated for each individual to quantify the relationship between each test individual and the members of their breed.

## Results

The MAF spectrum of the SNPs on the HD and F250 assays for the individuals that composed our reference panel is shown in Fig. 1, which also displays data for the SNP50 assay for comparison. The SNP50 and HD assays have similar MAF spectra and include mostly common variants. In addition, the HD assay has an increased density of variants with a MAF ranging from 0.025 to 0.075. However, the F250 assay has a much higher proportion of SNPs with a MAF lower than 0.1, which is more similar to the site frequency spectrum of variants identified from genome resequencing [38].

### Imputation accuracy metrics

**Fig. 2:**
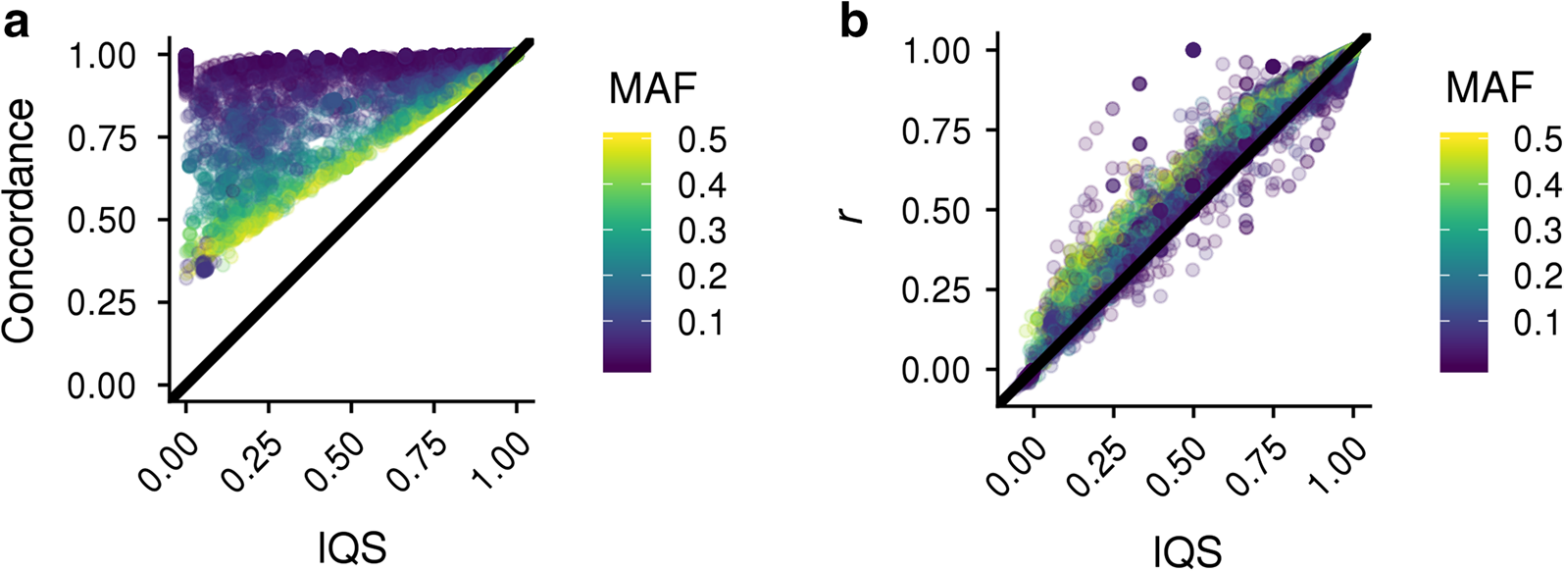
The imputation quality statistic (IQS) compared to concordance rate and correlation as measures of imputation accuracy. Three imputation accuracy measures calculated for the TAUR dataset. **a** concordance, and **b** Pearson correlation over-estimate imputation accuracies compared to the imputation quality statistic (IQS) resulting in bias and a false high imputation accuracy

Numerous statistics have been used to evaluate imputation quality. We compared two widely-used statistics [concordance rate and Pearson’s correlation (*r*)] with the imputation quality score (IQS), a metric that has been used in several human studies, but not in livestock [35, 39]. We tested each of these metrics on the TAUR dataset at both the level of variants and individuals. For variants, IQS were lower than concordance rates, particularly at lower MAF (Fig. 2a). In the TAUR dataset, IQS scores were lower than their corresponding *r* values for 81% of cases (Fig. 2b). At moderate to high MAF, these metrics generally agreed with each other. However, when MAF were lower than 0.1, both Pearson’s correlations and IQS penalized more heavily the imputation errors made for rare variants and resulted in lower averages and larger variances compared to concordance rates.

Since IQS is a metric for assessing variant accuracy, we used error type/count and Pearson’s correlation (*r*) between observed and imputed genotypes to determine the impacts of different intrinsic and extrinsic factors on accuracy of imputation for each individual. Individual *r* values using raw and centered genotypes were highly correlated (Pearson’s *r* = 0.9993 and Spearman’s *r* = 0.9998). Since these values were so highly correlated, we report only individual correlations calculated from the raw genotype values, hereafter. For our 307 test animals, individual *r* ranged from 0.7466 to 0.9993, but 267 of these individuals had *r* higher than 0.990. In addition to characterizing these metrics, we also identified the type of error (complete discordance, false heterozygote, or false homozygote) that occurred on a SNP and individual basis. Individuals with the lowest *r* values (< 0.85) tended to have significantly more false heterozygote errors than false homozygote errors (p = 1.475 × 10^−5^), whereas well imputed animals showed no significant difference (p = 0.7891).

### Comparing multi-breed and within-breed imputation reference panels

**Fig. 3:**
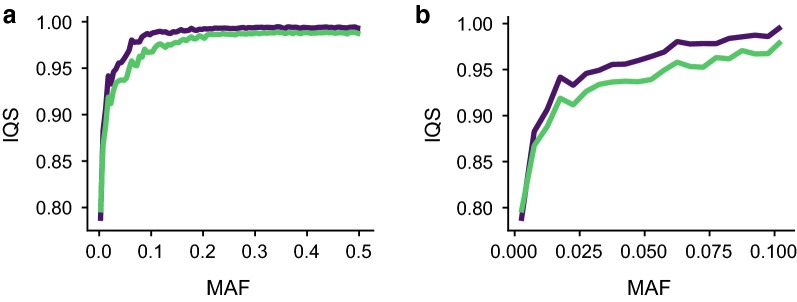
The composite reference panel improves per-variant imputation accuracies, particularly for rare variants. Imputation quality statistics when using breed-specific (green) and composite (purple) reference panels for 850 K imputation in the Gelbvieh (GEL) dataset across the MAF spectrum (**a**), and at low MAF (**b**)

We used 50 Gelbvieh animals with both HD and F250 genotypes that were masked to SNP50 genotype density to compare the accuracy of imputation obtained when using a multi-breed composite reference (CR) or a single-breed reference (BR) panel when imputing to 850 K SNPs. Gelbvieh had the most complete genotypes of any open herdbook breed in our reference, making it a best-case scenario for breeds with mixed ancestry. Imputation with the breed-specific imputation panel had a mean IQS score of 0.982 (sd = 0.089). Because the breed-specific panel performed well, overall mean accuracy gains were modest but significant when using the composite panel (IQS mean = 0.990, sd = 0.073, paired T-test p < 2 × 10^−16^) (Fig. 3a and see Additional file 5: Figure S3a). In addition to an increase in mean accuracy, the per-SNP accuracy variance decreased significantly when using the CR compared to the BR reference panel (F-test p < 2 × 10^−16^). Of the 107,110 SNPs for which IQS changed when imputed against the different reference panels, 89,930 had an increased score with the CR panel (average IQS increase compared to BR = 0.0797), whereas only 15,349 (average IQS decrease compared to BR = 0.0603) had a decreased score. For these two sets of SNPs, the average magnitude of the accuracy increases was significantly greater for the CR panel than for the BR panel (p < 2 × 10^−16^).

The most substantial accuracy gains from the use of the CR panel were observed for low MAF variants (Fig. 3b and see Additional file 5: Figure S3b). Although accuracy gains were modest for variants with MAF higher than 0.1 (0.007 IQS increase), the increase in IQS for rare variants was 0.0182 when imputing with the CR panel. This increase in the quality of low MAF imputation was not detected when using concordance rate or *r* statistics (Table 3). Of the 122,288 markers that were not perfectly imputed using the BR panel, there was an increase in IQS of 0.059 (*r* increase 0.032) when imputed with the CR panel.

**Table 3:** Per-variant mean imputation accuracy measures by MAF for Gelbvieh individuals imputed using the breed reference (BR) and composite reference (CR) panels

MAF	BR GC^a^	CR GC^a^	BR *r*	CR *r*	BR IQS	CR IQS
> 0.00–0.05	0.999	0.999	0.984	0.982	0.910	0.926
> 0.05–0.10	0.997	0.998	0.985	0.99	0.959	0.979
> 0.10–0.15	0.996	0.998	0.986	0.992	0.974	0.989
> 0.15–0.20	0.995	0.997	0.988	0.993	0.982	0.991
> 0.20–0.25	0.994	0.997	0.990	0.995	0.986	0.993
> 0.25–0.30	0.994	0.997	0.990	0.995	0.987	0.993
> 0.30–0.35	0.994	0.997	0.991	0.996	0.988	0.994
> 0.35–0.40	0.993	0.997	0.992	0.996	0.988	0.994
> 0.40–0.45	0.993	0.996	0.992	0.996	0.988	0.994
> 0.45–0.50	0.993	0.996	0.992	0.996	0.988	0.994

^a^Genotype concordance

One concern with using a large multi-breed reference panel for imputation is that it may introduce variation that does not actually exist in the population being imputed. Individuals had significantly fewer false heterozygote errors when using the CR panel compared to the BR panel (paired T-test p = 0.0039). There were, on average, 733 fewer false heterozygote calls per individual when the CR panel was used.

**Fig. 4:**
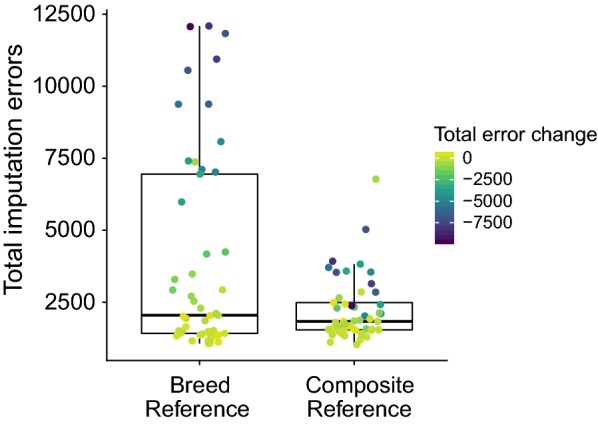
Improvements from the composite reference panel were greatest for individuals for which genotypes were poorly imputed. Comparing the total number of errors when imputing from SNP50 to 850 K in the Gelbvieh (GEL) dataset when using breed-specific vs. composite reference panels. Points are individuals, colored by the change in count of errors from the breed to composite reference panels

Whereas the per-variant increases in imputation accuracy were significant, the most substantial improvements in imputation accuracy due to the use of the CR panel were found for specific individuals. The mean individual *r* increased significantly from 0.9962 (s.d. = 0.0032) with the BR panel to 0.9979 (s.d. = 0.0010) with the CR panel (p = 0.0012). Animals that already had their genotypes accurately imputed using the BR panel did not show significant increases in accuracy with the CR panel. However, animals with the largest number of BR panel-induced imputation errors had much greater increases in accuracy when the CR panel was used (Fig. 4). The 14 individuals with more than 5000 total errors when the BR panel was used had, on average, 5522 fewer imputation errors (s.d. = 2361.33) when the CR panel was used for imputation. Conversely, the 35 individuals with less than 5000 imputation errors when the BR panel was used had only 209 fewer imputation errors, on average (s.d. = 609.80), when the CR panel was used for imputation.

Across the MAF spectrum, accuracies for the “one-round” imputation were consistently higher than those for the “two-round” method. However, the overall magnitudes of the differences were modest. The “one-round” imputation increased the overall accuracy of imputation by 0.000762 IQS units, and for low MAF variants by 0.00256 units. In the “one-round” imputation, the addition of imputed rare variants from the F250 into the combined reference also increased the imputation accuracy of rare variants that were exclusive to the HD panel. The HD markers with MAF lower than 0.05 that were imperfectly imputed using the “two-round” method had an average increase in IQS of 0.0846 when imputed by the “one-round” approach (Table 4). For the HD variants with moderate to high MAF, imputation accuracy increased slightly with the “one-round” compared with the “two-round” approach.

**Table 4:** The mean IQS by MAF for HD-specific markers that were imperfectly imputed using the “two-round” method

MAF	Number of SNPs	Two-round IQS	One-round IQS	IQS change
> 0.00–0.05	11,788	0.5453	0.6299	0.0846
> 0.05–0.10	18,095	0.9221	0.9330	0.0109
> 0.10–0.15	23,311	0.9724	0.9742	0.0017
> 0.15–0.20	29,222	0.9772	0.9784	0.0012
> 0.20–0.25	34,062	0.9803	0.9812	0.0009
> 0.25–0.30	39,948	0.9807	0.9815	0.0009
> 0.30–0.35	44,309	0.9815	0.9824	0.0009
> 0.35–0.40	47,657	0.9814	0.9822	0.0008
> 0.40–0.45	49,233	0.9815	0.9823	0.0008
> 0.45–0.50	50,908	0.9816	0.9823	0.0007

### Impact of the breed representation in the reference panel on imputation accuracy

**Fig. 5:**
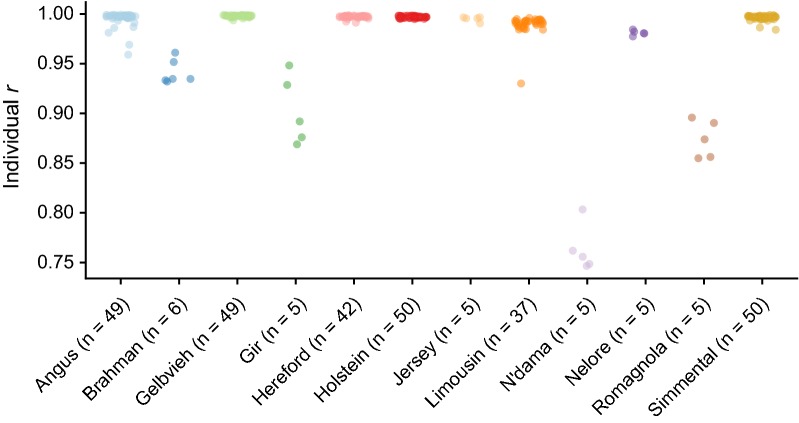
Per-individual accuracy by reported breed. Individual *r* by breed. Each point is an individual, colored by breed

Using individual imputation accuracy measures for 307 test animals, we identified the effects of an individual’s breed composition and of those breeds’ representations in the CR panel on individual imputation accuracy. Using the CR panel, individual *r* ranged from 0.747 to 0.999 while total imputation errors per individual ranged from 932 to 219,737. The accuracy of imputation was strongly related to an animal’s identified breed (Table 5). Individuals from breeds that were adequately represented in the CR panel (Angus, Gelbvieh, Hereford, Holstein, Jersey, Limousin, Nelore and Simmental, Table 2) were generally well imputed (median *r* = 0.997, range = [0.930, 0.999]) (Fig. 5). Gelbvieh individuals had the highest mean imputation accuracy (*r* = 0.998), which is likely due to the high proportion of Gelbvieh animals genotyped on both the F250 and HD in the reference panel. Gelbvieh comprised 10.66% of the reference panel individuals with complete 850 K genotypes, second only to Holstein (80.13% of total). Since HD markers represent the largest proportion of the 850 K SNP panel, individuals from breeds with large numbers of HD genotypes, but relatively few F250 genotypes, such as Nelore, were still imputed at high accuracy (median *r* = 0.981, range = [0.9774, 0.9844]). Individuals from breeds that were only sparsely represented in the CR panel (Brahman, Gir, N’Dama, and Romagnola) had decreased mean accuracies and increased per-animal imputation accuracy variances (mean *r* = 0.890, range = [0.747, 0.961]).

**Table 5:** Mean, minimum and maximum individual accuracies (*r*) by breed for the composite reference 850 K imputation

Breed	Mean	Min	Max
Gelbvieh	0.9979	0.9935	0.9989
Hereford	0.9971	0.9912	0.9988
Holstein	0.9969	0.9947	0.9984
Simmental	0.9963	0.9841	0.9990
Angus	0.9953	0.9590	0.9993
Jersey	0.9950	0.9905	0.9966
Limousin	0.9892	0.9300	0.9960
Nelore	0.9810	0.9774	0.9844
Brahman	0.9412	0.9320	0.9611
Gir	0.9027	0.8689	0.9482
Romagnola	0.8742	0.8549	0.8958
N’Dama	0.7632	0.7466	0.8033

**Fig. 6:**
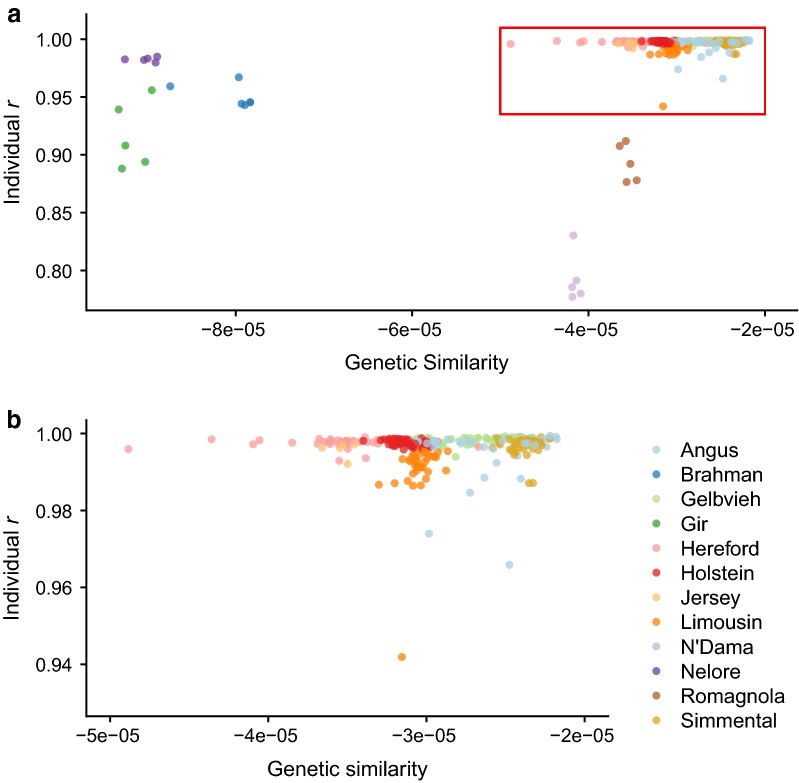
Relatedness to composite reference panel members is not a strong predictor of individual imputation accuracy. **a** Per-individual *r* for the entire testing dataset as a function of individual’s genetic similarity to the composite reference. **b** Zoom-in on high-imputation accuracy taurine individuals. Larger values indicate stronger relationships

We used a GRM that was created with observed genotypes from all reference and test individuals to determine if an individual’s genetic similarity to individuals in the CR panel was related to its imputation accuracy (Fig. 6). There was no direct relationship between an individual’s average relatedness to members of the CR panel and imputation accuracy. Rather, imputation accuracy was better predicted by the breed representation of the individuals in the CR panel. For example, individuals assigned by the CRUMBLER pipeline as Romagnola had relatively low imputation accuracies (mean individual *r* = 0.874, range = [0.8549, 0.8958]), although their genetic similarity values were comparable to those for the Hereford and Jersey samples. The low imputation accuracy for the Romagnola breed likely stems from the low representation of its haplotypes within the CR panel (15 HD and 8 F250 genotypes). We observed the opposite for Nelore; although the Nelore individuals were distantly related to the members of the CR panel as a whole, the larger number of samples contained in the reference panel (858 HD and 7 F250 genotypes) resulted in accurate imputation (mean individual *r* = 0.981). This was also observed for Gir, which is as diverged from taurines as the Nelore breed, but its reduced imputation accuracy was due to the presence of only 13 HD and nine F250 individuals in the CR panel. The average genetic relationship with individuals of the same breed in the CR panel had varying magnitudes of correlation with individual imputation accuracies, depending on the breed (see Additional file 6: Figure S4). Measures of genetic similarity and individual imputation accuracy were highly correlated in Brahman and Nellore (*r* = 0.940), negatively correlated in Gelbvieh (*r* = − 0.138), and moderately correlated in Angus and Holstein (*r* = 0.207 and 0.241, respectively).

### Impact of the starting assay number of markers on 850 K imputation

**Fig. 7:**
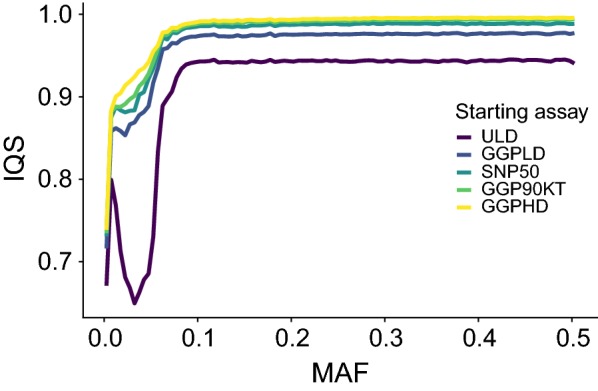
Effects of starting assay marker numbers on imputation accuracy across the MAF spectrum. Variant accuracy measures for 850 K imputation in the TAUR dataset based on five assays with different marker numbers. Binned mean IQS lines (per-variant accuracy) across the MAF spectrum

To test the impact of the starting assay number of markers on 850 K imputation accuracy, we used the TAUR dataset masked to represent the contents in markers of five common commercial assays. Each successive increase in assay marker number led to increases in imputation accuracy both overall and for low-MAF variants (Table 6 and Fig. 7). The largest increase in imputation accuracy came between the number of markers used in ULD and GGP-LD assays. Imputation accuracies from the ULD were exceptionally poor for low-MAF variants. Although the decline in IQS at low MAF was also observed for other assays, it was much greater for the ULD variants (0.1385 IQS decrease). At marker densities higher than that of the GGP-LD assay, increases in overall imputation accuracy were smaller (GGP-LD → SNP50 = 0.0133, SNP50 → GGP-90KT = 0.0051, and GGP-90KT → GGP-HD = 0.0036). Similar increases in accuracy were observed for low-MAF variants as the starting assay density increased (ULD → GGP-LD = 0.1249, GGP-LD → SNP50 = 0.0152, SNP50 → GGP-90KT = 0.0099, and GGP-90KT → GGP-HD = 0.0099).

**Table 6:** Per-variant mean and standard deviations for imputation quality statistic (IQS) for 850 K imputation in the TAUR dataset based on the starting assay density

Starting assay	Starting density	Mean IQS	SD IQS	Mean IQS (low MAF)^a^	SD IQS (low MAF)^a^
ULD	6394	0.9095	0.1766	0.7720	0.3503
GGP-LD	16,854	0.9612	0.1225	0.8969	0.2604
SNP50	44,366	0.9745	0.1154	0.9121	0.2468
GGP-90KT	70,581	0.9796	0.1104	0.9220	0.2402
GGP-HD	125,446	0.9832	0.1032	0.9319	0.2264

^a^Variants with minor allele frequencies < 0.1

**Fig. 8:**
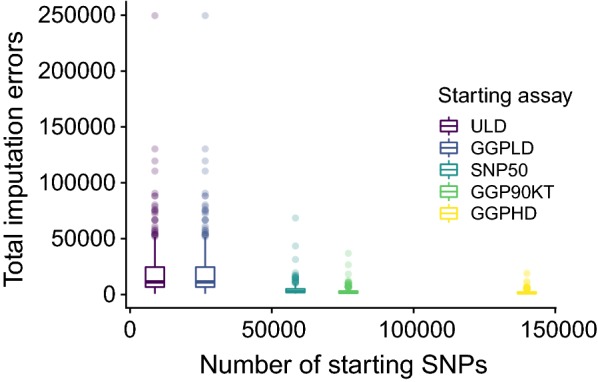
Impact of starting assay marker numbers on per-individual imputation accuracy. Per-individual accuracy measures for 850 K imputation in the TAUR dataset based on five starting assays differing in marker numbers. Boxplots for total imputation errors based on each starting assay marker number

Individual accuracies also increased as the starting assay number of markers increased. A one-way ANOVA using Tukey’s method for multiple comparisons indicated a significant difference in 850 K imputation accuracy between the ULD and GGP-LD (p = 9.05 × 10^−5^) assays, but not between the GGP-LD and SNP50 (p = 0.1486) assays (Fig. 8). There were no significant differences between the SNP50 and GGP-90KT or GGP-HD assays. However, the starting GGP-LD marker number had a significantly lower imputation accuracy compared to GGP-90KT (p = 0.0049). This suggests that imputation accuracy gains are minimal when the starting assay marker number is larger than 50,000 variants (Table 7).

**Table 7:** Per-individual *r* mean and standard deviation values for 850 K imputation based on starting assay density

Starting assay	Starting density	Mean *r*	SD *r*
ULD	6394	0.989	0.6070
GGP-LD	16,854	0.995	0.0486
SNP50	44,366	0.997	0.0314
GGP-90KT	70,581	0.998	0.0205
GGP-HD	125,446	0.999	0.0129

### Error profiles and regions of low imputation accuracy

**Fig. 9:**
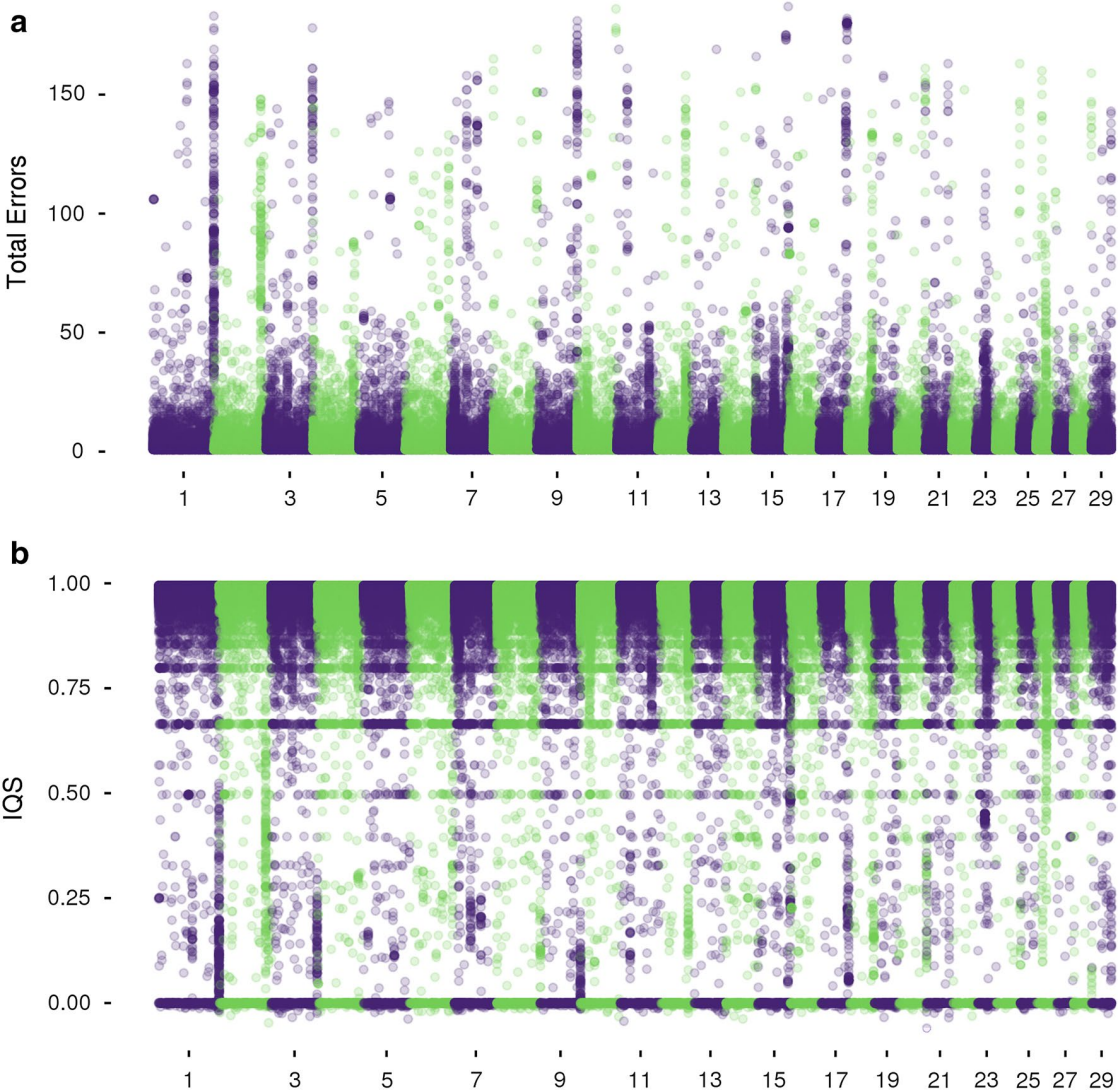
Regions with low imputation accuracy exist across the genome but represent only a small subset of the markers. Regions of low imputation accuracy using the TAUR dataset identified by total imputation errors (**a**), and IQS (**b**)

Using the imputation accuracy information for the TAUR dataset, we identified a number of genomic regions for which markers had a low imputation accuracy. Although most markers were accurately imputed, most chromosomes have at least one small region that contained poorly imputed markers (Fig. 9). The overall number of poorly imputed markers was quite small. Only 21,848 markers had an IQS lower than 0.8 (1.95% of imputed makers) (see Additional file 5: Figure S3a and S3b), and only 8963 markers had more than 10 imputation errors (1.07% of imputed markers). When using the IQS metric, we found that there are markers imputed with low accuracies on each chromosome, particularly low-MAF variants with relatively few errors (making IQS = 0) (Fig. 9a). However, both IQS and total error counts (Fig. 9b) reveal clusters of markers with a low imputation accuracy. Investigation of these regions indicated that the probe sequences for these variants had multiple equally likely matches to the genome, which indicates either that there were genome mis-assemblies or simply that the wrong location was chosen to represent the position of the marker. The latter can be easily rectified by changing the map files for these variants to reflect the correct alternate position.

## Discussion

### Imputation accuracy metrics

Most studies on imputation accuracy in livestock populations have used two methods to assess the adequacy of imputation: concordance rate, i.e. the proportion of correctly imputed genotypes, and the Pearson correlation (*r*) between observed and imputed genotypes. Although both statistics make sense at the level of individuals, their ability to identify markers for which genotypes are poorly imputed is not optimal, particularly for markers with a low MAF. Because our dataset contains a large proportion of rare variants (24.30% markers with MAF < 0.1), a statistic that more robustly represents the quality of imputation is essential. Using the IQS statistic, we show that *r* and especially concordance rate, overestimate the accuracy of imputation for low-MAF variants [35, 40]. In the GEL test data, 2070 variants with an average MAF of 0.040 had high concordance rates (0.97 average), but very low IQS scores (0.0). Unlike *r*, which requires that markers be variable in both the true and imputed datasets, IQS can be calculated for variants that are not variable in either the observed or imputed datasets. This provides a more complete view of the imputation accuracy at each locus, particularly for those with an extremely low MAF. This information is lost when using *r*, and imputation accuracies are grossly inflated if measured using concordance rate. That said, *r* and IQS are highly correlated (*r* = 0.9892) and provide equally useful diagnostics for imputation quality at most sites. We note that while we treated the HD and F250 genotype calls as being correct, a ~ 0.2% error rate is associated with these genotyping platforms [15] (see Additional file 1: Table S4).

### Impact of the F250 assay on imputation of rare variants

The F250 assay was designed to query genotypes at a large number of rare, potentially functional variants and is very gene-centric (i.e. they are not evenly spaced). Common variants were also included in the F250 assay design to allow for imputation and genomic prediction applications. The rare variants present on the F250 assay are important in the context of this work for two reasons. First, imputing an additional ~ 170,000 variants at the population level will increase researchers’ ability to refine GWAS signals and identify putative QTN due to increased marker density within QTL regions. Second, because the variants are very gene-centric, it is anticipated that the accuracy of imputation to the whole-genome sequence level will be improved within genic regions. The inclusion of rare variants will likely increase the imputation accuracy of other rare variants that are not directly assayed, as strong LD (*r*^*2*^) requires that allele frequencies at two markers be similar. In the absence of selection, rare variants are assumed to have been recently derived, and thus are likely in LD with other recently derived rare alleles [41]. By adding rare variants to our reference panel with the F250 assay and by genotyping a large number of individuals, we improve the imputation of rare variants that are not directly assayed by the F250. Although many individuals in our reference panel have only imputed F250 genotypes, their presence had a significant impact on the imputation accuracies of rare variants. Whereas at a reduced scale, our comparison of “one-round” vs. “two-round” imputation showed that leveraging rare F250 variants helped impute low-MAF variants that are only assayed by the HD assay (Table 4). We expect that these increases in imputation accuracy of rare variants that are achieved from the use of the F250 assay will be carried over to subsequent imputation to whole-genome sequence-level. The positive impact of the F250 assay on imputation of rare variants underscores the need for additional complete 850 K data in our reference panel (individuals genotyped with both the HD and F250 assays). The highest imputation accuracies were observed for breeds that had the largest numbers of complete 850 K genotypes because more of the haplotypic diversity in those breeds was directly captured in the reference panel.

### Multi-breed vs. within-breed imputation reference panels

Early imputation studies primarily concentrated on homogenous populations. When imputation is performed in closely related animals from breeds with small effective population sizes, such as Holstein [42, 43], highly accurate imputation can be achieved from using a relatively small set of reference genotypes. Recently, large numbers of genotypes have been produced using low-density assays in outbred animals, admixed individuals, from both registered and commercial populations. In conjunction, many animals from a wide range of breeds have now been genotyped on high-density assays such as HD and F250. By combining all available high-density genotypes into a single multi-breed composite reference panel, we found increased imputation accuracy across the MAF spectrum. Comparing the composite reference panel with a breed-specific reference panel, the most substantial increases occurred at the level of individuals. Genotypes for individuals that were accurately imputed using the breed reference panel saw no substantial increases in accuracy when imputed using the CR panel. However, individuals with poorly imputed genotypes using the BR panel had a substantial reduction in the number of imputation errors when imputed using the CR panel. The increased haplotypic diversity present in the composite reference panel improves the accuracy of imputation of introgressed haplotypes that are not present in a more limited breed-specific reference panel. It is important to be aware that in the context of routine genotyping and imputation, there is no a priori knowledge on which individuals may have poorly imputed genotypes. On the one hand, gains in accuracy from using the CR panel may be small in closed herdbook populations such as Holstein or Angus but they are unlikely to be worse than if the panel is restricted to a breed-specific reference. On the other hand, for open herdbook or composite breeds, increases in imputation accuracy are likely substantial. We did not detect an increase in false heterozygote or false homozygote genotype calls using the multi-breed reference panel, which suggests that the use of a CR panel does not introduce false variation into imputed genotypes at a higher rate than imputation using a within-breed reference panel. For breeds that are adequately represented in the CR panel, we found imputation accuracies (median *r* = 0.997) that were consistent with the error rates of the genotyping assays (see Additional file 1: Table S4), which suggests a near-perfect imputation process.

Previous work recommended the use of multi-breed reference panels for whole-genome sequence imputation [19, 44]. Our findings for high-density genotypes with an allele frequency spectrum similar to that of the genome sequence supports this finding and suggests that improvements in imputation accuracy for outbred and admixed populations will benefit from the sequencing and inclusion of diverse animals that will capture more of the haplotypic diversity that is found in cattle. Further improvements in accuracy could be obtained by removing Mendelian inconsistencies from the raw dataset that is used to create the CR panel, which was not performed for this study.

### Breed representation in the reference panel

An individual’s average relatedness to the entire CR panel was not a good predictor of imputation accuracy. Our multi-breed reference panel was heavily biased towards the most common and economically relevant American beef breeds but also had a diverse array of individuals from other breeds in varying numbers. We found that even low levels of admixture with breeds not adequately represented in the CR panel can lead to decreased imputation accuracies. Information on breed composition was valuable for identifying outlying individuals or breeds that, in theory, should have been accurately imputed. For example, the five individuals labeled as Angus with low imputation accuracies were found to be admixed with breeds that are not well represented in the CR panel (see Additional file 3: Table S6). Each of these individuals identified as Angus actually had relatively low proportions of Angus ancestry (0.107 to 0.532 Angus and Red Angus), and moderately high proportions of breeds sparsely represented in the CR panel. The most significant increases in imputation accuracy will likely come through the addition of high-density genotypes for breeds that are sparsely represented in our reference panel, and through the addition of more completely genotyped individuals, i.e., those with both HD and F250 genotypes. It is worth noting that the breed accuracies reported here for populations with a limited representation in our composite reference panel (Brahman, Gir, N’Dama, Romagnola) would have improved if we had not removed large proportions of each of the breeds to create the test set. We expect that the accuracies reported here are underestimated compared with those achieved by imputation against the full CR panel.

### Starting assay marker numbers

The starting assay marker number had a significant impact on the accuracy of imputation to 850 K. In agreement with the conclusions on LD of the Bovine HapMap project, we found that approximately 50 K SNPs are needed to impute to 850 K with high accuracy [45]. This observation likely has a larger impact on research applications that seek to identify QTN rather than applications that are targeted towards genomic prediction. At common allele frequencies (MAF > 0.1), IQS values were steady for all starting assay densities. The decline in imputation quality of rare variants (MAF < 0.1) relative to MAF was much more severe for low-density starting assays, particularly the ULD assay, than for higher density starting assays. When starting array densities are increased above 50 K SNPs, significant gains in imputation accuracy will come almost exclusively from improved imputation at rare variants. There is a large number of individuals that have been genotyped with assays with small numbers of common markers (< 10,000 markers) and these individuals can be accurately imputed to ~ 50 K common markers [41, 42]. Studies that impute from these densities to 850 K and whole-genome sequence should expect significantly more errors. If the aim is to perform both genomic predictions and downstream causal variant discovery, via imputation, our recommendation is to genotype new individuals with an assay density of ~ 50,000 SNPs.

## Conclusions

We conclude that, in diverse samples, as seen in typical beef cattle populations, a multi-breed phasing and imputation panel will provide the highest imputation accuracies. Individuals that have a moderately represented ancestry in the reference panel will have genotypes accurately imputed. Imputation accuracies were highest for rare variants when using the composite reference panel. The addition of rare variants from the F250 assay increased the imputation accuracy of rare variants in the HD assay. The addition of a large number of individuals that are genotyped for rare variants will likely improve imputation of rare variants to the sequence level. We confirm that for imputation to 850 K, gains in accuracy reach a plateau as the starting assay marker number exceeds 50 K SNPs. We identified a small subset of SNPs with poor imputation accuracies, most of which seem to be caused by location errors of probe sequences that can be corrected. The largest gains in imputation accuracy are expected to come from the addition of individuals with complete (HD and F250) genotypes, with the largest gains coming from modest increases in the numbers of individuals from the less well-represented breeds. Imputation accuracies for the breeds that are adequately represented in the multi-breed composite-reference panel when the starting assay comprises at least 50 K SNPs should approach accuracies of 1.0 minus the genotyping assay error rate. We anticipate that the CR panel presented here will serve as a foundation reference panel, on which the global cattle community can build to further increase the accuracy of genotype imputation.

## Supplementary information

**Additional file 1.** Additional information describing the design of the GGP-F250 assay [15, 46–50]. **Table S1.** Number of variants present on the GGP-F250 assay for each of the design waves. Description: The wave refers to the order in which candidate variants were selected to be added to the manifest in order to use 250 K beads. Designed: number of variants present in the manifest submitted for synthesis, Final Manifest: number of variants in the final delivered manifest, Failed Synthesis: number of variants that failed oligo synthesis, Filtered: number of variants that passed filtering based on automated and/or manual clustering using 18,684 individuals, Final List: number of variants in the final marker list that were used to produce genotypes, Variable: number of variants with at least one alternate allele observed in 18,684 individuals. **Table S2.** Description of abbreviations used in Table S1 as filtering criteria. **Table S3.** Animals by breed used to develop the Illumina cluster file for genotyping. Removed denotes samples that were excluded from clustering due to low call rate. **Table S4.** Genotype reproducibility for samples genotyped twice. Variation in the total number of compared genotypes is due to the individual sample call rates on each assay.

**Additional file 2: Figure S1.** Schematic representation of genotype masking for imputation testing.

**Additional file 3: Table S5.** Shared variants between analyzed assays. Counts of shared, unfiltered markers between assays used in this analysis. **Table S6.** Outlier samples identified as Angus and their CRUMBLER-estimated breed composition. Bold values represent the largest values that sum to at least 75% of an individual’s total breed composition. The percentage of individuals from each breed with HD genotypes in the CR panel is indicated.

**Additional file 4: Figure S2.** Schematic representation of “one-round” vs. “two-round” imputation. Description: Dotted lines represent imputation. In “one-round” imputation (a), HD and F250 reference samples are cross-imputed to create a partially imputed composite reference panel (1). This is followed by a single round of imputation of low-density genotypes using the CR panel (2). For “two-round” imputation (b), two rounds of imputation occur: first from low-density to HD (1) and then from HD to 850 K (2).

**Additional file 5: Figure S3.** Imputation quality metrics when using breed-specific (green) and composite (purple) reference panels for 850 K imputation in the GEL dataset across the entire MAF spectrum (a), and at low MAF (b). Points are individual variants.

**Additional file 6: Figure S4.** Impact of genetic similarity to the reference on imputation accuracy. Description: Genetic similarity is the mean genomic relationship between testing individual and reference individuals with > 50% ancestry of the same breed. Gelbvieh testing individuals (a) are colored by the change in *r* when using CR versus the BR. (b–d) show *r* vs. genetic similarity for Angus, Holstein, and Brahman/Nelore respectively.

## Data Availability

The datasets analyzed during the current study are not publicly available because the data originated from a variety of sources with different ownership and sharing agreements. However, data are available from the corresponding author on reasonable request. Please contact authors regarding data availability or to receive imputed genotypes from our pipeline.
