# Supplementary material for: A multi-breed reference panel and additional rare variants maximize imputation accuracy in cattle
Source: Genet Sel Evol. 2019 Dec 26;51:77. doi: 10.1186/s12711-019-0519-x (PMC6933688; doi:10.1186/s12711-019-0519-x)
Supplement: Supplementary file 1 — Additional file 1. Additional information describing the design of the GGP-F250 assay [15, 46–50]. Table S1. Number of variants present on the GGP-F250 assay for each of the design waves. Description: The wave refers to the order in which candidate variants were selected to be added to the manifest in order to use 250 K beads. Designed: number of variants present in the manifest submitted for synthesis, Final Manifest: number of variants in the final delivered manifest, Failed Synthesis: number of variants that failed oligo synthesis, Filtered: number of variants that passed filtering based on automated and/or manual clustering using 18,684 individuals, Final List: number of variants in the final marker list that were used to produce genotypes, Variable: number of variants with at least one alternate allele observed in 18,684 individuals. Table S2. Description of abbreviations used in Table S1 as filtering criteria. Table S3. Animals by breed used to develop the Illumina cluster file for genotyping. Removed denotes samples that were excluded from clustering due to low call rate. Table S4. Genotype reproducibility for samples genotyped twice. Variation in the total number of compared genotypes is due to the individual sample call rates on each assay. [file 12711_2019_519_MOESM1_ESM.docx]

**Additional Information Describing the Design of the GGP-F250 Assay**

**Motivation:**

We were involved in a number of projects that all involved whole genome sequence (WGS) or RNA-seq data generation with the objective of identifying variants more predictive of phenotypes. One of the main objectives of one project was to sequence a number of bulls to try to identify putative lethal alleles and build a low-density genotyping assay to test these variants in a large sample to identify those variants that were never observed as homozygous and thus based upon their frequency within the sample could be filtered to identify candidate lethal alleles. Two further projects required additional genotyping to fine-map QTL for feed efficiency and risk of bovine respiratory disease. Given each of these projects’ need for additional genotyping, and an independent project to develop a “functional assay,” we built a single 250K assay in an attempt to simultaneously meet the needs of all of these projects. Given the very specific research requirements of these projects, we anticipated a higher than normal failure/false-positive rate for the loci placed on the assay. In order to make the assay of more general use and also have the ability to integrate this assay with other commercial assays via imputation, we incorporated 33,730 well-spaced and highly informative loci into the design.

**Data Sources:**

A total of 244 *Bos taurus* genomes were whole genome sequenced (referred to as Taurus genomic). The majority of the sequencing was performed at the University of Missouri DNA Core Facility. Each animal had two libraries prepared with average fragment lengths of 350 bp and 450 bp and were sequenced to ~20-24X coverage on Illumina Hi-Seq 2000/2500 instruments. All WGS data are deposited in BioProjects PRJNA343262 and PRJNA294306 at NCBI. Additional sequence data were obtained from Genome Canada (Paul Stothard) and USDA/BARC (George Liu) and included in the analysis for variant discovery. A total of 16 *Bos indicus* individuals (referred to as Indicus genomic) were also sequenced and used for variant discovery. Additionally, more than 150 *Bos taurus* individuals with RNA-seq data from 1-6 tissues per animal (30-80M reads/tissue) were used for variant discovery. RNA-seq data were deposited in BioProjects PRJNA294306, PRJNA272725 and PRJNA522422 at NCBI. Finally, we included variant calls from Run 5 of the 1000 Bulls Project (1K Bulls) [[46]](https://paperpile.com/c/7Csomo/0psnK).

**Data Processing:**

All of the sequence data (except for the 1K Bulls) was processed using a pipeline developed at the University of Missouri that included adapter trimming using a custom Perl script followed by error correction using MaSuRCA v2.1.1 [[47]](https://paperpile.com/c/7Csomo/ugwid). Briefly, for each animal, all FASTQ files were processed through MaSuRCA to error correct the reads producing a pe.cor.fa interleaved FASTA file. A custom Perl script then parsed the error corrected reads into forward and reverse sequence read files removing any reads less than 35 bp in length. The error corrected FASTA files were then aligned to the UMD3.1 reference genome [[48]](https://paperpile.com/c/7Csomo/0Lnou) which was augmented with ChrY from the Btau4.1 assembly [[49]](https://paperpile.com/c/7Csomo/hzM5b) using NextGENe v2.3.4 (SoftGenetics, State College, PA) which also performed variant calling and variant annotation. The resulting variant calls per animal were imported into a Postgres database for querying.

**Variant Selection:** There were three primary sources of variants: 1) Taurus genomic, 2) Taurus RNA-seq and 3) 1K Bulls. Variants were annotated and included in the assay design in waves similar to the approach of Matukumalli et al. [[15]](https://paperpile.com/c/7Csomo/Scuby) without regard to genomic distribution. Variants of primary interest were those that affected the amino acid sequence of a gene such as non-synonymous, frameshift, premature stop codon, etc., as these variants are more likely to have a functional impact. Other classes of variants included those that fell within genomic regions identified as conserved non-coding elements [[50]](https://paperpile.com/c/7Csomo/bJeoB) or within QTL regions from the aforementioned research projects.

Of particular interest were variants observed in at least two individuals for which no homozygotes were observed among the sequenced individuals and that lay within the annotation boundaries of a gene, but that were not necessarily coding. For each of these primary pools, variants were selected that had not been selected by the previous wave as a candidate for inclusion in the assay. A variant was considered validated if it was observed in multiple sequence data sources, which included the three described data sources, was located on an available genotyping assay or within dbSNP.

**Assay Design Tool:** All of the candidate variants (N=2,388,925) were submitted to the Illumina Assay Design Tool (ADT) to obtain designability scores. Importantly, when specifying the flanking sequence for a variant, the IUPAC code for any flanking variant with an estimated MAF ≥ 0.15 was included in the flanking sequence. This allowed ADT to place the probe on the side of the variant not containing a known high-frequency variant, if possible. For a subset of variants (N=193,506), ADT was run a second time only specifying flanking variants within ± 12 bp of the target variant (referred to as redesign below) in order to “rescue” some of the potentially more impactful candidates.

**Final Design:** After obtaining design scores, variant selection was performed in waves as shown in **Table S3** based on a number of criteria described in **Table S4**. Referring to **Table S3**, the different waves in which variants were added to the design manifest is shown in the first column. Note that there are missing waves due to the need to remove entire waves in order to reach the 250K bead limit for assay design.

**Final Genotypes:** All genotyping of the GGP-F250 assay was performed at GeneSeek (Lincoln, NE) and intensity files were transferred to the University of Missouri. A total of 18,786 animals from multiple breeds (**Table S5**) were included in a single Illumina GenomeStudio project and were clustered using default parameters. Manual curation of the cluster file was performed by filtering on various quality metrics and manually inspecting the clusters after removing 102 samples that had low call rate based on initial clustering. A total of 14,566 markers were flagged for exclusion due to various reasons such as low call rate, high Mendelian error rate, visible presence of null alleles or failure to cluster. The resulting cluster file produced genotypes for 206,549 loci, of which 175,135 were variable in the samples used for clustering. A total of 52 samples were run in duplicate with an average concordance rate between replicates of 99.83% indicating highly reproducible genotypes (**Table S6**). A total of 1,297 samples that were genotyped on both the BovineSNP50 and GGP-F250 showed an average concordance rate of 99.98% at an average of 21,220 informative loci indicating high reproducibility of genotypes across assays (data not shown). Overall, there was a high conversion rate to variable markers for the waves which had higher confidence during marker selection and a much lower conversion rate for waves that had lower confidence such as those with the “NoHOM“ or “not validated” tags.

**Table S1**. Number of variants present on the GGP-F250 assay for each of the design waves. The wave refers to the order in which candidate variants were selected to be added to the manifest in order to use 250K beads. Designed: number of variants present in the manifest submitted for synthesis, Final Manifest: number of variants in the final delivered manifest, Failed Synthesis: number of variants that failed oligo synthesis, Filtered: number of variants that passed filtering based on automated and/or manual clustering using 18,684 individuals, Final List: number of variants in the final marker list that were used to produce genotypes, Variable: number of variants with at least one alternate allele observed in 18,684 individuals.

| **Wave** | **Description** | **Designed** | **Final Manifest** | **Failed Synthesis** | **Filtered** | **Final List** | **Variable** | **Percent Converted** | **Percent Variable** |
| --- | --- | --- | --- | --- | --- | --- | --- | --- | --- |
| 0 | Imputation content | 33,730 | 33,062 | 668 | 333 | 32,729 | 32,428 | 98.99 | 99.08 |
| 1 | Variable ANG\|Coding\|NoHOM\|Validated | 16,157 | 15,719 | 438 | 1,060 | 14,659 | 9,066 | 93.26 | 61.85 |
| 2 | Variable ANG\|Coding\|NoHOM\|NOT validated | 14,052 | 13,663 | 389 | 291 | 13,372 | 5,032 | 97.87 | 37.63 |
| 3 | Coding\|Validated | 71,246 | 69,219 | 2,027 | 5,163 | 64,056 | 57,573 | 92.54 | 89.88 |
| 4 | Splice\|Validated | 7,594 | 7,330 | 264 | 1,961 | 5,369 | 3,324 | 73.25 | 61.91 |
| 5 | FS or In-Frame\|Validated | 524 | 513 | 11 | 149 | 364 | 197 | 70.96 | 54.12 |
| 6 | ncRNA\|Validated | 1,196 | 1,159 | 37 | 77 | 1,082 | 1,028 | 93.36 | 95.01 |
| 7 | Variable ANG\|Genomic\|Splice ±8\|NoHOM\|Validated | 872 | 845 | 27 | 38 | 807 | 618 | 95.50 | 76.58 |
| 8 | Variable ANG\|Genomic\|Splice ±8\|Validated | 4,209 | 4,064 | 145 | 188 | 3,876 | 3,796 | 95.37 | 97.94 |
| 9 | Genomic\|Splice ±8\|Validated\|MAF genomic OR TBG ≥0.01 | 2,200 | 2,132 | 68 | 25 | 2,107 | 2,065 | 98.83 | 98.01 |
| 10 | In-Frame\|NOT validated | 246 | 235 | 11 | 12 | 223 | 64 | 94.89 | 28.70 |
| 11 | FS | 504 | 491 | 13 | 51 | 440 | 235 | 89.61 | 53.41 |
| 13 | 5' UTR with highest priority and highest MAF per gene (MAF ≥0.05) | 10,794 | 10,505 | 289 | 578 | 9,927 | 9,751 | 94.50 | 98.23 |
| 14 | 3' UTR with highest priority and highest MAF per gene (MAF ≥0.05) | 8,551 | 8,309 | 242 | 485 | 7,824 | 7,617 | 94.16 | 97.35 |
| 15 | Highest MAF for genes without selected variant\|Validated | 5,597 | 5,421 | 176 | 233 | 5,188 | 4,845 | 95.70 | 93.39 |
| 17 | RD\|Variable ANG\|Coding\|NoHOM\|Validated | 4,001 | 3,863 | 138 | 357 | 3,506 | 1,767 | 90.76 | 50.40 |
| 18 | RD\|Variable ANG\|Coding\|NoHOM\|NOT validated | 4,635 | 4,501 | 134 | 115 | 4,386 | 1,612 | 97.45 | 36.75 |
| 19 | RD\|Coding\|Validated | 7,819 | 7,589 | 230 | 852 | 6,737 | 5,714 | 88.77 | 84.82 |
| 20 | RD\|Splice\|Validated | 1,028 | 993 | 35 | 184 | 809 | 428 | 81.47 | 52.90 |
| 21 | RD\|FS or In-Frame\|Validated | 233 | 225 | 8 | 111 | 114 | 50 | 50.67 | 43.86 |
| 22 | RD\|ncRNA\|Validated | 189 | 186 | 3 | 30 | 156 | 140 | 83.87 | 89.74 |
| 23 | RD\|Variable ANG\|Genomic\|Splice ±8\|NoHOM\|Validated | 192 | 182 | 10 | 19 | 163 | 102 | 89.56 | 62.58 |
| 24 | RD\|Variable ANG\|Genomic\|Splice ±8\|Validated | 773 | 743 | 30 | 81 | 662 | 645 | 89.10 | 97.43 |
| 25 | RD\|Genomic\|Splice ±8\|Validated\|MAF genomic OR TBG ≥0.01 | 266 | 261 | 5 | 4 | 257 | 244 | 98.47 | 94.94 |
| 26 | RD\|In-Frame\|NOT validated | 63 | 58 | 5 | 3 | 55 | 23 | 94.83 | 41.82 |
| 27 | RD\|FS | 166 | 163 | 3 | 27 | 136 | 62 | 83.44 | 45.59 |
| 28 | RD\|Coding or Splice\|NOT validated\|MAF≥0.01 | 905 | 884 | 21 | 33 | 851 | 332 | 96.27 | 39.01 |
| 29 | Variable ANG\|Coding\|NoHOM\|Validated\|RELAXED | 14 | 14 | 0 | 5 | 9 | 6 | 64.29 | 66.67 |
| 31 | Coding\|Validated\| RELAXED | 1,978 | 1,929 | 49 | 349 | 1,580 | 1,492 | 81.91 | 94.43 |
| 33 | FS or In-Frame\|Validated\| RELAXED | 63 | 60 | 3 | 23 | 37 | 23 | 61.67 | 62.16 |
| 34 | ncRNA\|Validated\| RELAXED | 182 | 180 | 2 | 44 | 136 | 127 | 75.56 | 93.38 |
| 35 | Variable ANG\|Genomic\|Splice ±8\|NoHOM\|Validated\| RELAXED | 164 | 155 | 9 | 18 | 137 | 104 | 88.39 | 75.91 |
| 36 | Variable ANG\|Genomic\|Splice ±8\|Validated\| RELAXED | 520 | 505 | 15 | 65 | 440 | 413 | 87.13 | 93.86 |
| 37 | Genomic\|Splice ±8\|Validated\|MAF genomic OR TBG ≥0.01\| RELAXED | 207 | 201 | 6 | 13 | 188 | 184 | 93.53 | 97.87 |
| 40 | CNE\|Variable ANG\|No HOM | 73 | 69 | 4 | 3 | 66 | 58 | 95.65 | 87.88 |
| 41 | QTL\|CNE | 1,777 | 1,739 | 38 | 19 | 1,720 | 1,720 | 98.91 | 100.00 |
| 42 | QTL\|1bead | 15,789 | 15,444 | 345 | 1,143 | 14,301 | 14,299 | 92.60 | 99.99 |
| 44 | QTL\|1bead RELAXED | 7,714 | 7,521 | 193 | 268 | 7,253 | 7,228 | 96.44 | 99.66 |
| 50 | Coding\|2 variants | 815 | 790 | 25 | 154 | 636 | 532 | 80.51 | 83.65 |
| 51 | CNE filler | 195 | 193 | 2 | 2 | 191 | 191 | 98.96 | 100.00 |
|  | Total | 227,233 | 221,115 | 6,118 | 14,566 | 206,549 | 175,135 | 93.41 | 84.79 |

**Table S2**. Description of abbreviations used in Table S1 as filtering criteria.

| **Abbreviation** | **Description** |
| --- | --- |
| Imputation | High MAF variants selected with uniform spacing from existing assays for imputation between assays |
| Variable ANG | Variable within sequenced Angus individuals |
| Coding | Within the coding region of a gene |
| NoHOM | No homozygous individuals observed |
| Validated | Variant present in more than one independent data source |
| NOT validated | Variant present in only a single data source |
| Splice | Directly affects an annotated splice site |
| Splice ±8 | Variant within ±8 bp of an annotated splice site |
| FS | Indel that produces a frame-shift within a CDS |
| In-Frame | Indel that produces an in-frame variant within a CDS |
| ncRNA | Within the annotated region of a non-coding RNA |
| Genomic | Not within an annotated gene region, intergenic |
| 5’ or 3’ UTR | Within the annotated 5’ or 3’ untranslated region of a gene |
| TBG | 1000 Bull Genomes data minor allele frequency ≥ 0.01 |
| Highest MAF for genes without selected variant | For all annotated genes that had not previously had a variant included we chose the variant with the highest MAF within the boundary of the gene to tag that gene |
| RD | Redesigned with only variants ± 12 bp of the target variant included in flanking sequence |
| RELAXED | Lower design score criteria between 0.5 and 0.6. All other waves used design score >0.60 |
| CNE | Within the boundary of a Conserved Noncoding Element |
| QTL | Within the boundary of an identified QTL |

**Table S3.** Animals by breed used to develop the Illumina cluster file for genotyping. Removed denotes samples that were excluded from clustering due to low call rate.

| Breed | Samples | Avg. Call Rate |
| --- | --- | --- |
| Angus | 12,309 | 0.9883 |
| Holstein | 2,016 | 0.9920 |
| Red Angus | 1,333 | 0.9735 |
| Crossbred | 1,096 | 0.9841 |
| Hereford | 950 | 0.9935 |
| Gelbvieh | 308 | 0.9956 |
| Simmental | 275 | 0.9730 |
| Limousin | 267 | 0.9857 |
| Charolais | 20 | 0.9953 |
| Brahman | 14 | 0.9845 |
| Gir | 11 | 0.9827 |
| Santa Gertrudis | 11 | 0.9912 |
| Chirikof Island Cattle | 10 | 0.8853 |
| Jersey | 10 | 0.9849 |
| Nelore | 10 | 0.9639 |
| Piedmontese | 9 | 0.9915 |
| N'Dama | 8 | 0.9952 |
| Romagnola | 8 | 0.9953 |
| Braunvieh | 7 | 0.9933 |
| Guernsey | 7 | 0.9968 |
| Beefmaster | 3 | 0.9915 |
| Sheko | 2 | 0.9880 |
| Removed | 102 | 0.7179 |

**Table S4**. Genotype reproducibility for samples genotyped twice. Variation in total number of compared genotypes is due to the individual sample call rates on each assay.

| Sample 1 | Sample 2 | Same | Different | Total | Concordance % |
| --- | --- | --- | --- | --- | --- |
| 1291075851 | 1291075852 | 181,006 | 3,647 | 184,653 | 99.0076 |
| 1291075701 | 1291075702 | 176,047 | 3,311 | 179,358 | 99.0727 |
| 1291075961 | 1291075962 | 187,449 | 3,052 | 190,501 | 99.1957 |
| 1291073621 | 1291073622 | 188,183 | 2,885 | 191,068 | 99.2422 |
| 1291066901 | 1291066902 | 187,439 | 1,998 | 189,437 | 99.4713 |
| 1291075761 | 1291075762 | 184,393 | 1,957 | 186,350 | 99.4735 |
| 1020894921 | 1020894922 | 192,975 | 1,884 | 194,859 | 99.5154 |
| 1291075983 | 1291075981 | 179,012 | 1,745 | 180,757 | 99.5161 |
| 1291075821 | 1291075822 | 183,555 | 1,759 | 185,314 | 99.5243 |
| 1291075871 | 1291075872 | 185,576 | 1,647 | 187,223 | 99.5592 |
| 1291067981 | 1291067982 | 191,052 | 1,666 | 192,718 | 99.5668 |
| 1020894691 | 1020894692 | 198,656 | 598 | 199,254 | 99.8498 |
| 1020895001 | 1020895002 | 200,548 | 571 | 201,119 | 99.8579 |
| 1020894661 | 1020894662 | 198,602 | 424 | 199,026 | 99.8934 |
| 1020894871 | 1020894872 | 199,874 | 426 | 200,300 | 99.8936 |
| 1020894781 | 1020894782 | 200,748 | 400 | 201,148 | 99.9005 |
| 1020894681 | 1020894682 | 201,628 | 339 | 201,967 | 99.9160 |
| 1020894631 | 1020894632 | 201,714 | 335 | 202,049 | 99.9171 |
| 1020894981 | 1020894982 | 202,048 | 298 | 202,346 | 99.9263 |
| 1020894731 | 1020894732 | 202,478 | 270 | 202,748 | 99.9334 |
| 1020894891 | 1020894892 | 203,107 | 234 | 203,341 | 99.9425 |
| 1020894941 | 1020894942 | 203,336 | 205 | 203,541 | 99.9496 |
| 1020894641 | 1020894642 | 203,282 | 197 | 203,479 | 99.9516 |
| 1020894901 | 1020894902 | 203,421 | 196 | 203,617 | 99.9519 |
| 1020894831 | 1020894832 | 202,788 | 192 | 202,980 | 99.9527 |
| 1020894651 | 1020894652 | 203,057 | 186 | 203,243 | 99.9542 |
| 1020894701 | 1020894702 | 203,289 | 169 | 203,458 | 99.9585 |
| 1020894861 | 1020894862 | 203,483 | 153 | 203,636 | 99.9624 |
| 1020895011 | 1020895012 | 203,921 | 147 | 204,068 | 99.9640 |
| 1020894851 | 1020894852 | 203,218 | 143 | 203,361 | 99.9648 |
| 1020894771 | 1020894772 | 202,979 | 134 | 203,113 | 99.9670 |
| 1020894821 | 1020894822 | 203,673 | 128 | 203,801 | 99.9686 |
| 1020894621 | 1020894622 | 203,283 | 127 | 203,410 | 99.9688 |
| 1291076014 | 1291076011 | 203,661 | 121 | 203,782 | 99.9703 |
| 1020894741 | 1020894742 | 204,027 | 117 | 204,144 | 99.9713 |
| 1020894791 | 1020894792 | 203,569 | 111 | 203,680 | 99.9728 |
| 1020894751 | 1020894752 | 204,404 | 104 | 204,508 | 99.9746 |
| 1020894841 | 1020894842 | 204,164 | 97 | 204,261 | 99.9763 |
| 1020894711 | 1020894712 | 203,930 | 95 | 204,025 | 99.9767 |
| 1020894801 | 1020894802 | 204,199 | 91 | 204,290 | 99.9777 |
| 1020894811 | 1020894812 | 204,306 | 90 | 204,396 | 99.9780 |
| 1020894671 | 1020894672 | 204,413 | 65 | 204,478 | 99.9841 |
| 1020894951 | 1020894952 | 204,699 | 61 | 204,760 | 99.9851 |
| 1020894931 | 1020894932 | 204,729 | 56 | 204,785 | 99.9863 |
| 1020894961 | 1020894962 | 204,618 | 55 | 204,673 | 99.9866 |
| 1020894991 | 1020894992 | 204,806 | 51 | 204,857 | 99.9876 |
| 1020895021 | 1020895022 | 205,118 | 44 | 205,162 | 99.9893 |
| 1030335131 | 1030335132 | 204,892 | 41 | 204,933 | 99.9900 |
| 5030334521 | 5030334522 | 202,859 | 31 | 202,890 | 99.9924 |
| 1020894761 | 1020894762 | 205,497 | 25 | 205,522 | 99.9939 |
| 1020894881 | 1020894882 | 205,598 | 11 | 205,609 | 99.9973 |
| 1000333501 | 1000333502 | 206,057 | 4 | 206,061 | 99.9990 |

**References:**

1[5. Matukumalli LK, Lawley CT, Schnabel RD, Taylor JF, Allan MF, Heaton MP, et al. Development and characterization of a high density SNP genotyping assay for cattle. PLoS One. 2009;4:e5350.](http://paperpile.com/b/7Csomo/Scuby)

[46. Hayes BJ, Daetwyler HD. 1000 Bull Genomes Project to Map Simple and Complex Genetic Traits in Cattle: Applications and Outcomes. Annu Rev Anim Biosci [Internet]. 2018; Available from:](http://paperpile.com/b/7Csomo/0psnK) <http://dx.doi.org/10.1146/annurev-animal-020518-115024>

[47. Zimin AV, Marçais G, Puiu D, Roberts M, Salzberg SL, Yorke JA. The MaSuRCA genome assembler. Bioinformatics. 2013;29:2669–77.](http://paperpile.com/b/7Csomo/ugwid)

[48. Zimin AV, Delcher AL, Florea L, Kelley DR, Schatz MC, Puiu D, et al. A whole-genome assembly of the domestic cow, Bos taurus. Genome Biol. 2009;10:R42.](http://paperpile.com/b/7Csomo/0Lnou)

[49. Bovine Genome Sequencing and Analysis Consortium, Elsik CG, Tellam RL, Worley KC, Gibbs RA, Muzny DM, et al. The genome sequence of taurine cattle: a window to ruminant biology and evolution. Science. 2009;324:522–8.](http://paperpile.com/b/7Csomo/hzM5b)

[50. Farré M, Kim J, Proskuryakova AA, Zhang Y, Kulemzina AI, Li Q, et al. Evolution of gene regulation in ruminants differs between evolutionary breakpoint regions and homologous synteny blocks. Genome Res. 2019;29:576–89.](http://paperpile.com/b/7Csomo/bJeoB)
