# Supplementary material for: A multi-breed reference panel and additional rare variants maximize imputation accuracy in cattle
Source: Genet Sel Evol. 2019 Dec 26;51:77. doi: 10.1186/s12711-019-0519-x (PMC6933688; doi:10.1186/s12711-019-0519-x)
Supplement: Supplementary file 2 — Additional file 2: Figure S1. Schematic representation of genotype masking for imputation testing. [file 12711_2019_519_MOESM2_ESM.pdf]

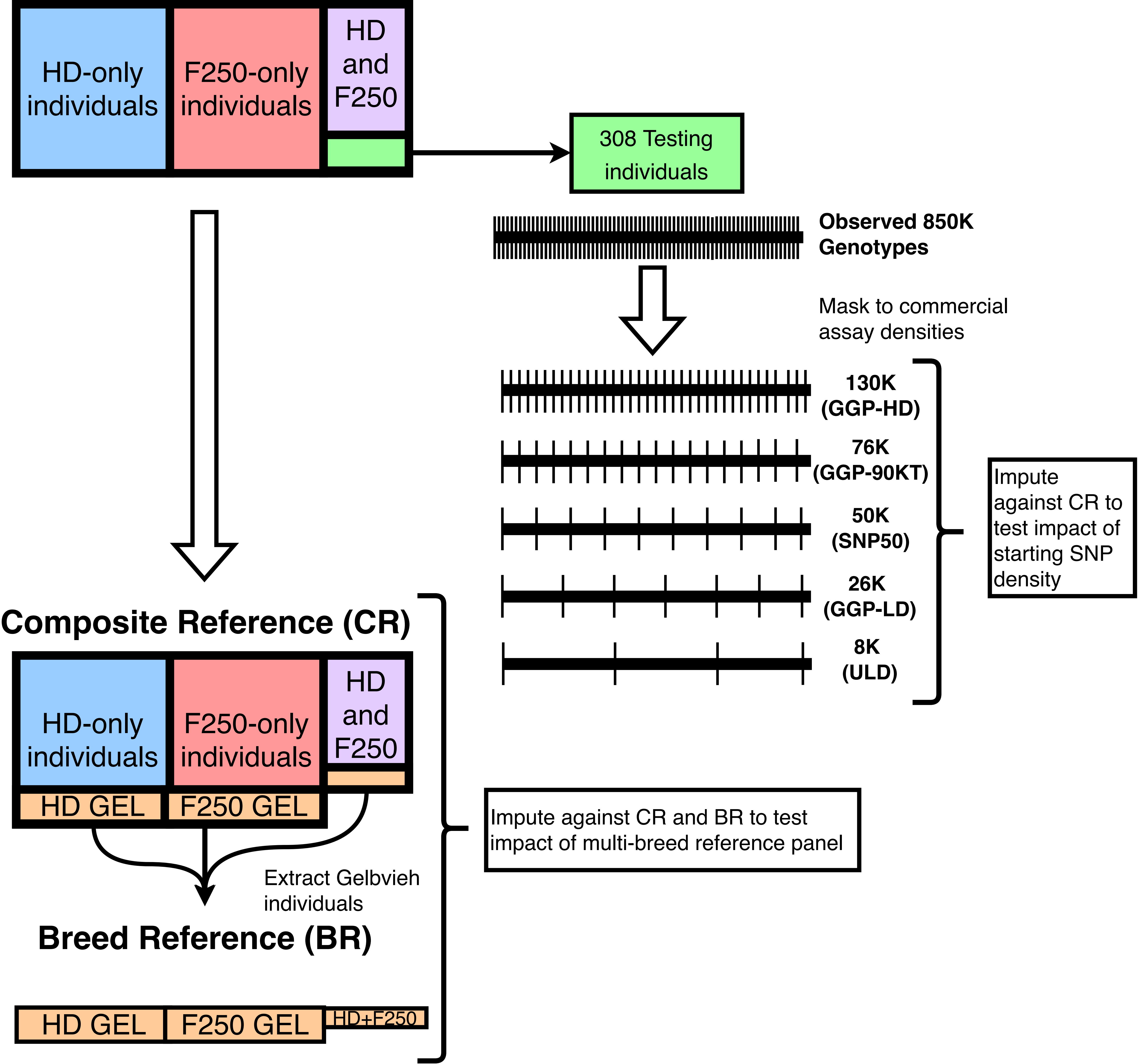
HD-only individuals
F250-only individuals
HD and F250
308 Testing individuals
Observed 850K Genotypes
Mask to commercial assay densities
130K (GGP-HD)
76K (GGP-90KT)
50K (SNP50)
26K (GGP-LD)
8K (ULD)
Impute against CR to test impact of starting SNP density
Composite Reference (CR)
HD-only individuals
F250-only individuals
HD and F250
HD GEL
F250 GEL
Extract Gelbvieh individuals
Breed Reference (BR)
HD GEL
F250 GEL
HD+F250
Impute against CR and BR to test impact of multi-breed reference panel
