# Supplementary material for: A multi-breed reference panel and additional rare variants maximize imputation accuracy in cattle
Source: Genet Sel Evol. 2019 Dec 26;51:77. doi: 10.1186/s12711-019-0519-x (PMC6933688; doi:10.1186/s12711-019-0519-x)
Supplement: Supplementary file 3 — Additional file 3: Table S5. Shared variants between analyzed assays. Counts of shared, unfiltered markers between assays used in this analysis. Table S6. Outlier samples identified as Angus and their CRUMBLER-estimated breed composition. Bold values represent the largest values that sum to at least 75% of an individual’s total breed composition. The percentage of individuals from each breed with HD genotypes in the CR panel is indicated. [file 12711_2019_519_MOESM3_ESM.docx]

**Table S5. Shared variants between analyzed assays.** Counts of shared, unfiltered markers between assays used in this analysis.

|  | **HD** | **F250** | **GGPHD** | **GGP90KT** | **SNP50** | **GGPLD** | **ULD** |
| --- | --- | --- | --- | --- | --- | --- | --- |
| **HD** | 777,962 | 37,841 | 134,599 | 74,524 | 50,665 | 25,693 | 8,280 |
| **F250** |  | 227,234 | 31,156 | 19,176 | 22,199 | 18,774 | 8,070 |
| **GGPHD** |  |  | 139,977 | 73,341 | 42,975 | 25,121 | 8,151 |
| **GGP90KT** |  |  |  | 76,999 | 28,952 | 14,240 | 7,948 |
| **SNP50** |  |  |  |  | 58,336 | 8,607 | 8,034 |
| **GGPLD** |  |  |  |  |  | 26,504 | 8,320 |
| **ULD** |  |  |  |  |  |  | 8,762 |

**Table S6.** Outlier samples identified as Angus and their CRUMBLER estimated breed composition. Bolded values represent the largest values that sum to at least 75% of an individual’s total breed composition. The percentage of individuals from each breed with HD genotypes in the CR panel is indicated.

|  |  | **Outlier #1** | **Outlier #2** | **Outlier #3** | **Outlier #4** | **Outlier #5** |
| --- | --- | --- | --- | --- | --- | --- |
| **Total Imputation Errors** |  | 43,536 | 31,412 | 19,588 | 14,718 | 14,506 |
| **R^2^** |  | 0.959 | 0.969 | 0.981 | 0.986 | 0.987 |
|  | **CR HD%** |  |  |  |  |  |
| **Angus** | 21.47 | **0.317** | **0.076** | **0.174** | **0.309** | **0.18** |
| **Brahman/Nelore** | 9.14 | **0.059** | 0 | 0 | 0.062 | 0.029 |
| **Braunvieh** | 0.00 | 0.033 | **0.283** | **0.069** | 0 | 0.003 |
| **Brown Swiss** | 0.16 | 0 | 0 | 0.003 | 0 | **0.049** |
| **Charolais** | 1.30 | 0.047 | 0.056 | **0.091** | **0.074** | 0.002 |
| **Gelbvieh** | 2.75 | 0 | **0.265** | **0.109** | **0.099** | 0 |
| **Guernsey** | 0.00 | 0.024 | 0.016 | 0.035 | 0.065 | 0.041 |
| **Hereford** | 5.91 | 0.026 | 0 | 0.055 | 0.001 | 0.022 |
| **Holstein** | 32.92 | **0.089** | 0 | 0.03 | 0.028 | 0.004 |
| **Jersey** | 0.22 | **0.065** | 0.015 | 0.007 | 0.003 | 0 |
| **Limousin** | 2.23 | 0.038 | **0.148** | **0.207** | 0.03 | 0.038 |
| **N'Dama** | 0.07 | **0.063** | 0.008 | 0 | 0 | 0.011 |
| **Red Angus** | 2.63 | **0.089** | 0.031 | 0.053 | **0.223** | 0.034 |
| **Romagnola** | 0.11 | 0.031 | 0.025 | 0 | 0.027 | 0.019 |
| **Shorthorn** | 1.41 | **0.088** | 0.039 | 0.064 | **0.077** | 0.042 |
| **Simmental** | 4.43 | 0.032 | 0.037 | **0.086** | 0 | **0.526** |
| **Wagyu** | 0.20 | 0 | 0 | 0.017 | 0.003 | 0 |
