# Supplementary figures and images for: A multi-breed reference panel and additional rare variants maximize imputation accuracy in cattle
Source: Genet Sel Evol. 2019 Dec 26;51:77. doi: 10.1186/s12711-019-0519-x (PMC6933688; doi:10.1186/s12711-019-0519-x)

## One-round imputation

1)

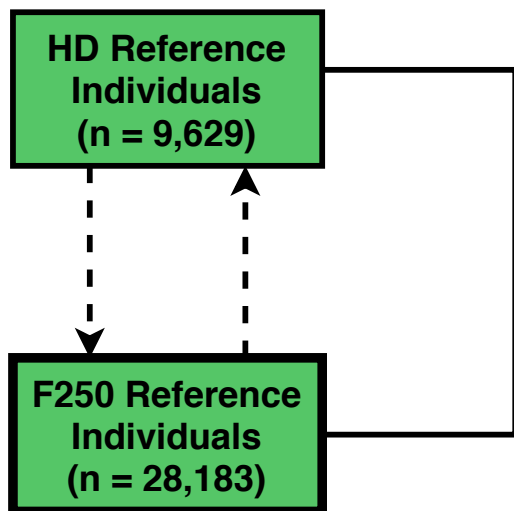

2)

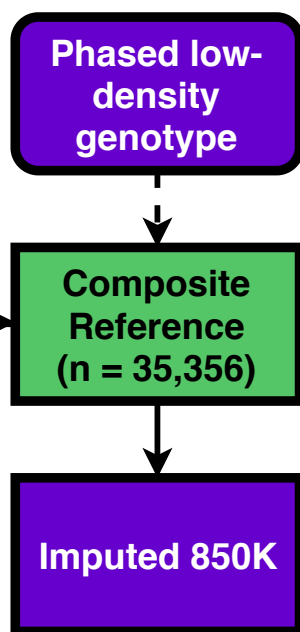

## Two-round imputation

1)

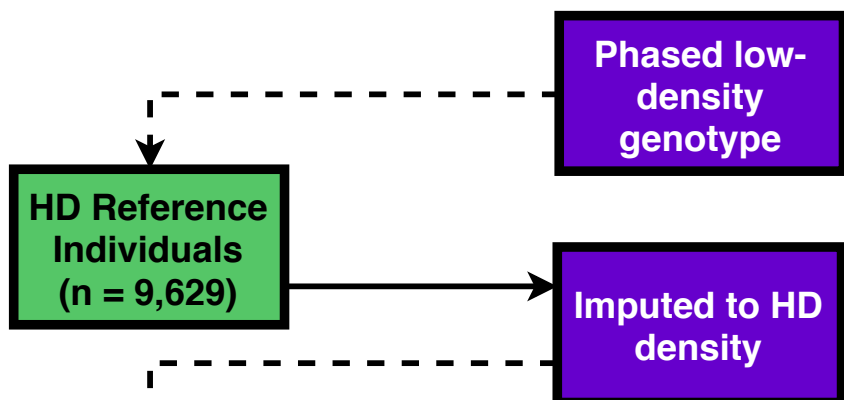

2)

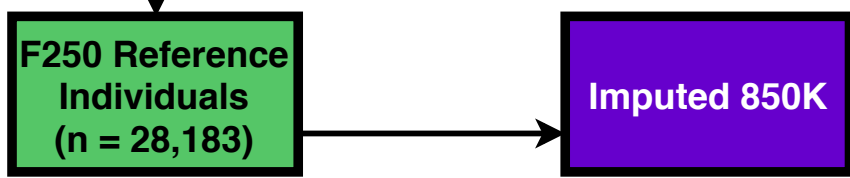

Supplement: Supplementary file 4 — Additional file 4: Figure S2. Schematic representation of “one-round” vs. “two-round” imputation. Description: Dotted lines represent imputation. In “one-round” imputation (a), HD and F250 reference samples are cross-imputed to create a partially imputed composite reference panel (1). This is followed by a single round of imputation of low-density genotypes using the CR panel (2). For “two-round” imputation (b), two rounds of imputation occur: first from low-density to HD (1) and then from HD to 850 K (2). [file 12711_2019_519_MOESM4_ESM.pdf]

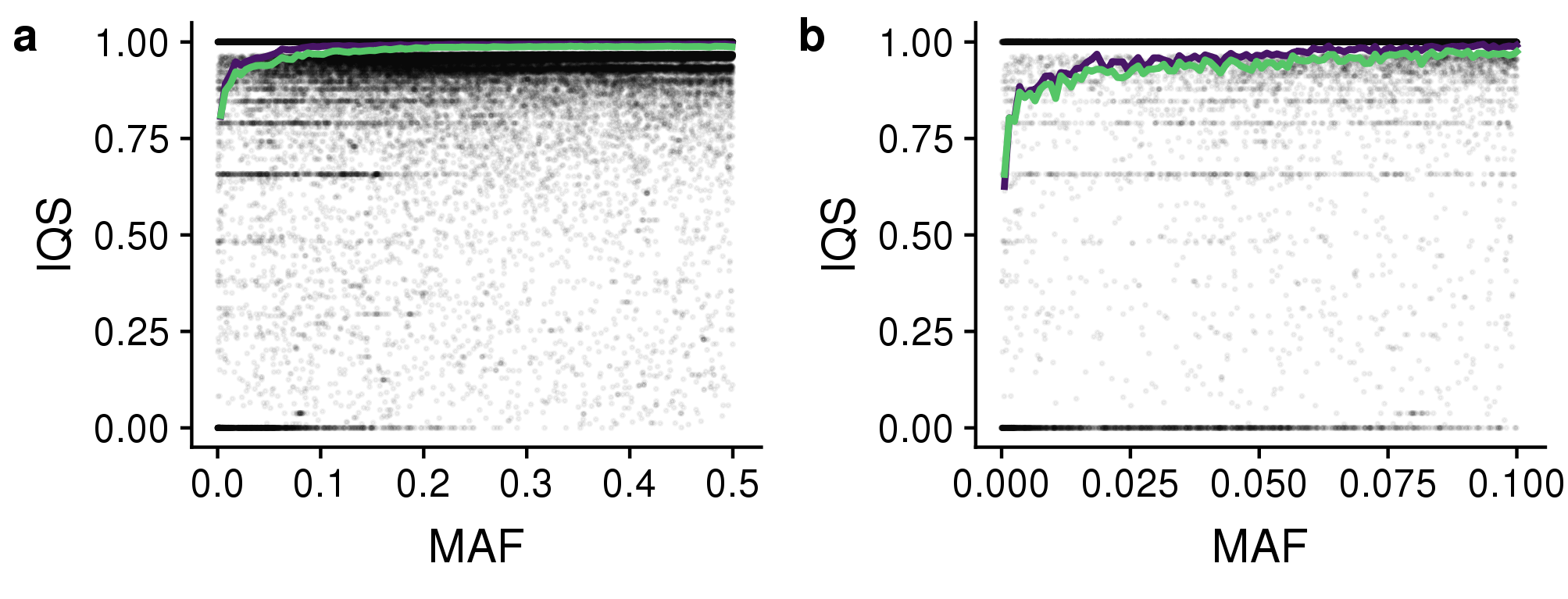

Supplement: Supplementary file 5 — Additional file 5: Figure S3. Imputation quality metrics when using breed-specific (green) and composite (purple) reference panels for 850 K imputation in the GEL dataset across the entire MAF spectrum (a), and at low MAF (b). Points are individual variants. [file 12711_2019_519_MOESM5_ESM.png]

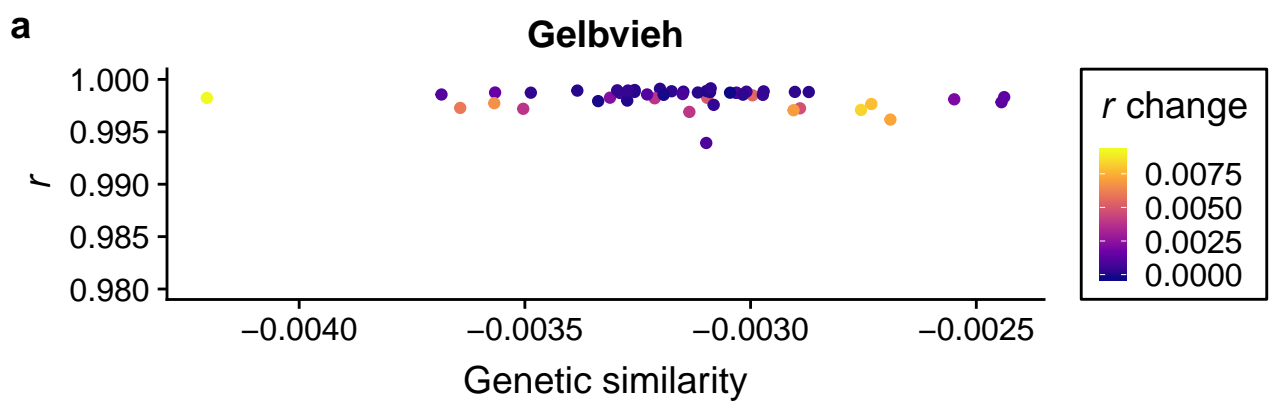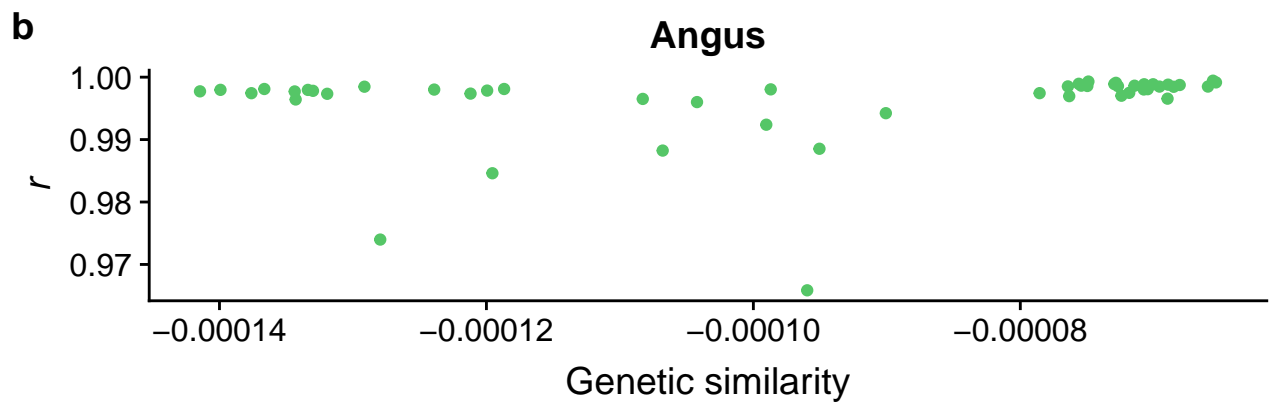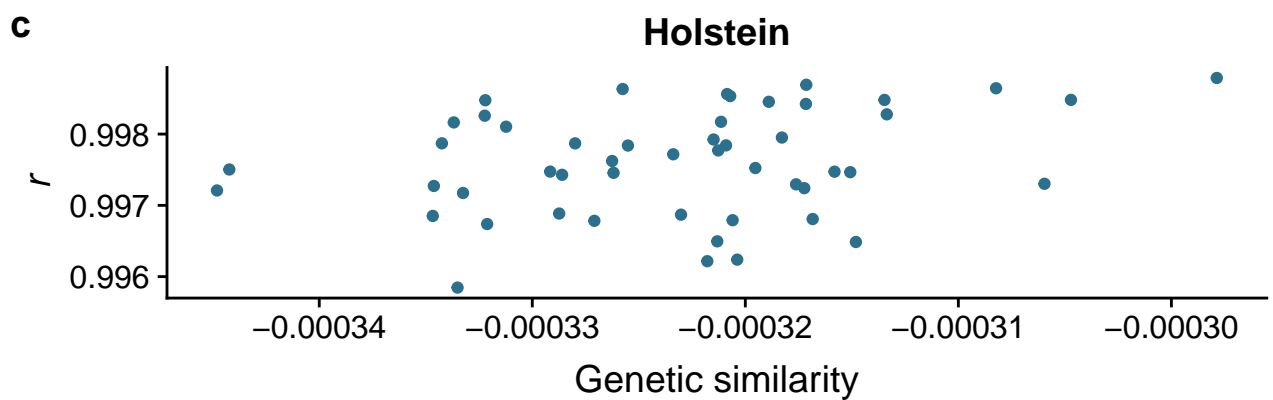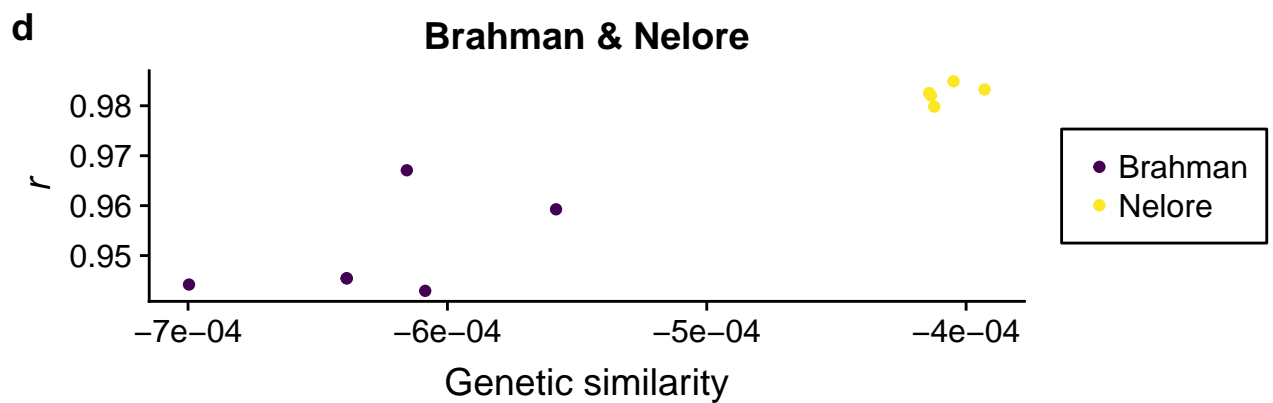

Supplement: Supplementary file 6 — Additional file 6: Figure S4. Impact of genetic similarity to the reference on imputation accuracy. Description: Genetic similarity is the mean genomic relationship between testing individual and reference individuals with > 50% ancestry of the same breed. Gelbvieh testing individuals (a) are colored by the change in r when using CR versus the BR. (b–d) show r vs. genetic similarity for Angus, Holstein, and Brahman/Nelore respectively. [file 12711_2019_519_MOESM6_ESM.pdf]
